# Spatio‐Temporal Variation in Diet Among Age and Sex Cohorts of a Model Generalist Bird Species, the Great Tit *Parus major* : New Insights Revealed by DNA Metabarcoding

**DOI:** 10.1002/ece3.71565

**Published:** 2025-07-14

**Authors:** J. R. Coomes, J. P. Cuff, M. S. Reichert, G. L. Davidson, W. O. C. Symondson, J. L. Quinn

**Affiliations:** ^1^ School of Biological, Earth and Environmental Sciences University College Cork Cork Ireland; ^2^ School of Natural and Environmental Sciences Newcastle University Newcastle‐upon‐Tyne UK; ^3^ Department of Integrative Biology Oklahoma State University Stillwater Oklahoma USA; ^4^ School of Biological Sciences University of East Anglia Norwich UK; ^5^ School of Biosciences Cardiff University Cardiff UK

**Keywords:** dietary variation, molecular dietary analysis, passerine, winter moth

## Abstract

Dietary variation among cohorts can have a major impact on how populations adapt to environmental variation. Although variation in diet between cohorts and across habitats has been studied in many taxa, this is not true for most birds, especially smaller generalist passerines whose feeding habits are predominantly cryptic. Here we used DNA metabarcoding with next‐generation sequencing to assess spatio‐temporal dietary variation among age and sex cohorts of the great tit *Parus major* , a model species in avian ecology. Most dietary species were rare but nevertheless collectively made up 30% of the diet, as expected of a generalist. Winter moth *Operophtera brumata* , a major focus in tit breeding phenology research, was the most prevalent dietary item, but the next ten most prevalent Lepidopterans were collectively four times more important. There was considerable variation in dietary richness and composition among seasons and years. In winter, natural plant and invertebrate species were extensively represented in the diet, despite the constant availability of supplemental food. Diet composition varied with woodland type: in conifer woodlands, birds fed on species adapted to conifer plantations, as expected, but they also fed on many species adapted to deciduous species. In winter, birds in conifers used peanut feeders more than they did in mixed woodlands where beech was more prevalent in the diet. In winter, first‐year birds consumed more invertebrate species than adults, presumably because they were less selective, and beech (*Fagus*) was almost twice as prevalent in first‐year diet. Our results suggest considerable spatio‐temporal variation in diet and variation among cohorts, and provide insight into the diet of a key model species in avian ecology. Such variation is rarely considered even though it is likely to have important consequences for our understanding of how populations respond to environmental change.

## Graphical abstract

- Identifying the sources of intraspecific dietary variation is central to understanding how populations adapt to environmental variation, and yet these sources are poorly understood in a key model species in avian ecology, the great tit.
- Here we use DNA metabarcoding to describe the diet of great tits at an unprecedented level of detail and reveal differences among age classes and sexes, likely reflecting differences in foraging modes and microhabitat use.
- We also show considerable variation in dietary richness and composition among seasons, years, and habitat types, indicating that we need to broaden our focus in order to understand drivers of population change.

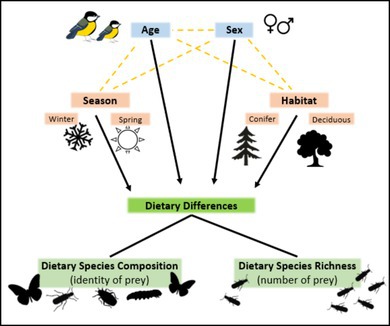

## 1 Introduction

Dietary generalism is common and allows animals to exploit many different food sources and habitats. Although generalism might at first suggest little variation between individuals, divergence can arise through a wide variety of intrinsic and extrinsic mechanisms. Diet can vary with age (Polis 1984; Ebenman 1987; Jones et al. 2020), perhaps because of increased foraging experience (Estes et al. 2003; Daunt et al. 2007; Fayet et al. 2015), changes in energetic requirements from growth to reproduction (Werner and Gilliam 1984; Munn and Dawson 2003), or changes in competitive ability (van Horne 1982; Polis 1984). Dietary differences can also occur among the sexes (Shine 1989; Clarke et al. 1998; Vasey 2002; Kamilar and Poekmpner 2008; Ratcliffe et al. 2013), and be driven by competition (Mason 1977), sexual size dimorphism (Birks and Dunstone 1985; Rose 1994), and different reproductive needs (Harrison 1983; Vasey 2002). Environmental factors, including seasonal effects (Betts 1955) and habitat variation, affect differences in diet (Kemenes and Nechay 1990; Newsome et al. 2015). Ultimately, population‐level effects also play a role since preferred prey become scarce when population size increases, potentially forcing less competitive individuals to select alternative prey items (Robinson and Wilson 1998; Svanbäck and Persson 2004; Svanbäck and Bolnick 2005, 2007).

Whatever the mechanism, identifying the sources of intraspecific dietary differences is central to understanding how populations adapt to environmental variation. Dietary differences are often unknown because identifying which food items are eaten can be challenging (Alberdi et al. 2019; Cuff, Windsor, et al. 2022). This is especially true in species that feed on small items or in ways that make direct observation difficult, leading to biases in our knowledge of diet variation. DNA metabarcoding can overcome many of the challenges presented by traditional, non‐molecular methods of dietary analysis, including invasive sampling (Betts 1955; van Balen 1973), detection biases (Moreby and Stoate 2000) and very coarse taxonomic resolution (Betts 1955; Bibby and Thomas 1985; Cramp and Perrins 1993). Undigested DNA in faeces is easily obtained non‐invasively and, at least in Europe, dietary metabarcoding can provide high taxonomic resolution because reference data are available for a relatively large proportion of invertebrate species in central databanks such as GenBank and BOLD (Barcode of Life Data Systems; barcodinglife.org) (Ratnasingham and Hebert 2007; Taberlet, Coissac, Hajibabaei, et al. 2012; Taberlet, Coissac, Pompanon, et al. 2012).

Here we use DNA metabarcoding to describe dietary variation at a taxonomic resolution that is unusually high for a member of the Passeriformes. For many decades, the great tit ( *Parus major* ) has been a key model species in avian ecology. In particular, the phenology of breeding in relation to the emergence of a single invertebrate species (the winter moth, *Operophtera brumata* ) has been a major research focus in the context of climate change (Visser and Holleman 2001; Vedder et al. 2013; Simmonds et al. 2020). However, the great tit is a generalist forager (Betts 1955; Pagani‐Núñez et al. 2015), with evidence for some degree of individual specialization (Pagani‐Núñez et al. 2015; Olivé‐Muñiz et al. 2021). Furthermore, the diet has been well documented, but primarily for nestlings and using traditional, non‐genetic, and morphology‐based methods (Betts 1955; Royama 1970; Töröck 1985; Naef‐Daenzer et al. 2000; Vel'ký et al. 2011). These methods can neglect many prey items; for example, hard‐part analyses fail to identify soft‐bodied prey, and direct observations do not capture nocturnal feeding (Cuff, Windsor, et al. 2022; Drake et al. 2023). Furthermore, it is difficult to characterise the diet of full‐grown birds because they feed away from the nest (Gibb 1954; Betts 1955). In this study, we investigate both richness (i.e., the number of species) and dietary composition of the species communities as a whole in the diet of full‐grown great tits.

**FIGURE 1:**
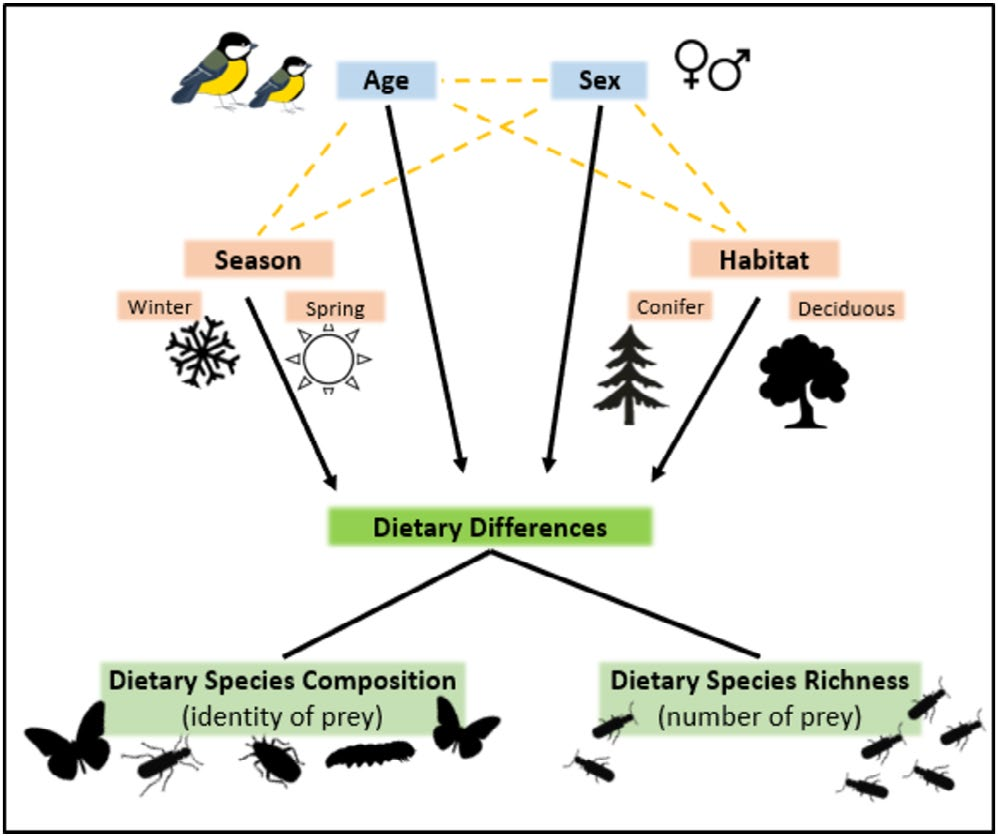
We investigated the influence of age, sex, season and habitat as main effects (black arrows), and in some cases as interactions (yellow dashed lines), on the diet of great tits. Two components of the diet were considered, composition (identity of species) and richness (number of species eaten) in two age categories (adults and first years), and in both sexes. Dietary invertebrates were identified in both winter and spring and dietary plants were investigated in the winter only.

Basic seasonal dietary differences have been documented in full‐grown great tits using gizzard analysis and observation (Gibb 1954; Betts 1955) but one of our key aims was to improve our knowledge of seasonal dietary differences using DNA metabarcoding. We expected that the higher taxonomic resolution of this method would reveal a greater diversity of animal and plant species in the diet. Additionally, dietary differences between habitats are very poorly characterized in the great tit and in most passerine species, so we examined diet across a landscape‐scale system of coniferous plantation and mixed‐deciduous woodland habitat fragments. In addition to examining habitat and seasonal (winter and spring) differences, we also explored differences among both age and sex cohorts, expecting divergence to arise through a variety of processes, including different dietary needs and competitive abilities (see Figure 1 for an illustration of the factors we investigated). We also expected differences among cohorts to be context dependent and more pronounced when environmental conditions were less favorable. Specifically, we expected more pronounced differences during winter than in spring, and in samples from mixed‐deciduous woodland rather than coniferous. Mixed‐deciduous habitats are less favorable than conifer because breeding density and competition is higher, leading to lower breeding success (O'Shea et al. 2018). Although we could not investigate individual specialization of diet due to a lack of repeated samples for each individual, we examine a range of factors that might lead to dietary divergence in this model species, which to our knowledge is among the few detailed dietary analyses of its kind in a passerine. Our results serve as a reminder that these diverse, usually unexplored sources of variation could change our understanding of how passerine populations respond to environmental change.

## 2 Materials and Methods

This study was conducted under licences from the Health Products Regulatory Authority (Project licence: AE19130/P017, Individual licence to JRC: AE19130/I250), the National Parks and Wildlife Service (Project licences: 004/2017, 001/2018, C02/2018; Individual licences to JRC: 70/2017, 11/2018) and the British Trust for Ornithology (individual ringing licences for JRC (C6597), SB and IHF). The research project received ethical approval from the Animal Welfare Body at University College Cork and was in accordance with the ASAB (Association for the Study of Animal Behaviour) Guidelines for the Treatment of Animals in Behavioural Research and Teaching.

### 2.1 Collection of Samples

Faecal samples were collected between May and June in 2017 and 2018 (*n* = 139), which we refer to as “spring”, and between November 2017 and February 2018 (*n* = 127), which we refer to as “winter”. We had six replicate study sites that were classified as conifer plantations (61 samples) and six sites that were classified as mixed‐deciduous woodland (205 samples) in the Bandon Valley, Ireland (see Table S1 and O'Shea et al. 2018 for study site details). In the mixed‐deciduous woodlands, the tree species commonly present were European beech (*Fagus sylvaticai*), English/pedunculate oak ( *Quercus robur* ), ash ( *Fraxinus excelsior* ), alder (*Alnus gultinosa*) and birch ( *Betula pendula* ). In the conifer woodlands, the species commonly present were Sitka spruce ( *Picea sitchensis* ), Lodgepole pine ( *Pinus contorta* ) and Norway spruce ( *Picea abies* ), among others (O'Shea et al. 2018). Our sites were relatively small woodland fragments (see Table S1 for size) set primarily within an intensive farming landscape with some human residential areas.

A trap‐door mechanism was used to trap full‐grown birds on the nest in spring, and mist nets with sunflower seeds and peanuts as bait were used to capture them in the winter. After capture by either method, birds were placed in coffee filters inside clean bags and were allowed time (not exceeding 30 min) to defecate. The coffee filter absorbed liquid urea, which can act as an inhibitor to amplification of DNA (Khan et al. 1991). Each filter was used once, and bird bags were cleaned between uses by washing in detergent. Following defecation, birds were fitted with a metal British Trust for Ornithology ring (if they had not been previously ringed for other ongoing studies) and were sexed and aged based on plumage (O'Connor 1985). Faecal samples were removed from bird bags via sterilised plastic rods and were transferred into collection tubes with 1 mL 100% ethanol. Care was taken not to let a sample come into contact with any other biological material. All equipment was sterilised between uses using bleach and ethanol. All samples were placed into a −20°C freezer at the end of the day and transferred to a −80°C freezer at the end of the season.

### 2.2 DNA Extraction

We established the invertebrate and plant species present in the faecal samples through identification of DNA sequences (see Figure S1) at Cardiff University, UK. First, DNA was extracted from the samples in batches of 18, and two extraction negatives were added per batch. In one instance, only eight samples were extracted in a batch and only a single extraction negative was included with that batch. In total, 264 samples were extracted: 137 spring samples (66 samples from 49 nests in 2017, and 71 samples from 49 nests in 2018) and 127 winter samples. The Qiagen Mini Stool Kit for DNA Extraction was used, together with the “DNA extraction from avian faeces stored in ethanol” protocol (Nicholls 2020), incorporating previous modifications (Shutt et al. 2020; Davies 2020) to deal with the small size and high levels of uric acid in avian faeces (see also Davies et al. 2022). For details of extraction protocol see the Appendix.

### 2.3 PCR Amplification to Establish Presence of DNA

Following extraction, target DNA regions were amplified using PCR (SimpliAmp Thermal Cycler or Applied Biosystems Robocycler Veriti). As with extractions, all equipment was autoclaved and/or cleaned with bleach and left under UV light for 15 min before PCR setup commenced.

We tested all samples for invertebrate DNA using primers that target the mitochondrial cytochrome c oxidase subunit I gene (COI) given that this is a standard for many groups of animals (Kress et al. 2015) including invertebrates (Hebert et al. 2003). The forward primer “mlCOIintF” (Leray et al. 2013) and the reverse “C1‐N‐2191/Nancy” (Simon et al. 1994) were used (Stockdale 2018) which target a 306 bp fragment of COI. This primer pair was selected to minimize amplification of host DNA and the data loss this represents (Cuff, Kitson, et al. 2022) and to achieve high taxonomic resolution. These primers have previously been tested on the diet of farmland thrushes (Stockdale 2018) and warbler species in the UK (Davies 2020) and across 18 orders and 50 families, most taxa were successfully amplified (Davies 2020); thus we are confident of their suitability for our study. The PCR conditions for COI were: initial denaturation at 95°C for 15 min, followed by 35 cycles of 94°C for 30 s, 55°C for 90 s, and 72°C for 90 s, and a final extension at 72°C for 10 min. In addition to the insect component of the diet, we also analyzed the plant DNA present, but only in the winter samples as we expected that in spring, great tits would be feeding almost exclusively on invertebrates. To amplify plant DNA, the “UniPlant” ITS2 (second internal transcribed spacer) primers (Moorhouse‐Gann et al. 2018) were used which target a short (160–320 bp) region. The PCR conditions for these reactions were identical for those above, but with 40 cycles rather than 35, an annealing temperature and duration of 56°C (58°C during validation, but reduced due to the melting temperature changing with the addition of tags) and 30 s, respectively, and an elongation time of 60 s.

Initial 5 μL PCRs were carried out to validate the success of DNA extraction. In each 5 μL PCR, 2 μL of the extracted DNA was included initially, alongside 2.5 μL Multiplex Master Mix (Qiagen), 0.1 μL of each of the 10 μM forward and reverse primers (final concentration = 0.2 μM) and the remaining volume made up using nuclease‐free water. The DNA extract volume was subsequently decreased to 1 μL for validation reactions of some samples to retain ample volume for sequencing in later steps, and the difference made up with nuclease‐free water. Following successful validation reactions, 25 μL PCRs were carried out, which consisted of 12.5 μL of Multiplex Master Mix (Qiagen), 5 μL of DNA, 2.5 μL each of the 2 μM forward and reverse primers (final concentration = 0.2 μM) and the remaining volume of nuclease‐free water. To the COI PCRs, we added 0.25 μL of 0.05 μg/mL BSA (Bovine serum albumin; Yu and Morrison 2004) to COI reactions, which improved amplification success.

Following PCR, gel electrophoresis was used to confirm successful amplification with a 2% agarose gel containing 1 μL of SyberSafe DNA dye. For samples in which DNA amplification was not successful, PCR was repeated once, and if amplification failed a second time, these samples were excluded from further work. Of 391 reactions (including both plant and invertebrate analyses across the 264 samples), 356 (91%) were successfully amplified. PCR primer sequences contained molecular identifier (MID) tags which allowed all samples to be pooled together and to be identified in downstream bioinformatic analyses by their unique combination of tags. Three PCR negative controls (nuclease‐free water in place of DNA) and two positive controls (PCR primer‐compatible DNA extracted from species that would not be consumed by the birds: whiteleg shrimp, *Penaeus vannamei* , and common mussel, *Mytilus edulis* , for COI, and two species of native Mauritian plant, *Dombeya mauritiana* and *Dodonaea viscosa* , for ITS2) were included in every PCR plate, alongside five extraction negatives (to include all of them across the PCR plates).

The concentration (ng/μL) of the target amplicon of the MID‐tagged PCR products was determined by high‐resolution capillary electrophoresis on a QIAxcel Advanced II (Qiagen). A high‐resolution DNA cartridge was used for the initial validation PCRs, and a DNA Fast Analysis cartridge was used subsequently. Failed PCRs were re‐run (up to a maximum of three times) with different tagged‐primer combinations in case tags affected PCR success. Tagged PCRs were successful for 88% (314 of 356) of reactions.

### 2.4 Pooling, Clean‐Up, Library Prep and Sequencing

Tagged PCR products were pooled together by PCR plate using the concentrations from the Qiaxcel to achieve approximate equimolarity. Samples that required pooling of more than 25 μL to be equimolar or which had a starting concentration of less than 2 ng/μL were considered failed reactions and were thus discarded. For plates in which the most concentrated sample was substantially more concentrated than most other samples, we diluted those samples with nuclease‐free water to facilitate accurate pooling of more samples. Extraction and PCR negatives were pooled with a volume equal to the average pooled for their respective plate, and no more than 10% of each pool was comprised of negative controls. In total, 120 PCR products were pooled for ITS2 and 386 for COI (this includes 192 nestling samples which were used in a separate study). The final result was two pools for ITS2 (79 and 58 samples, including controls) and eight pools for COI (15–81 samples, including controls).

Pools were cleaned and prepared for Illumina sequencing. Solid phase reversible immobilization (SPRI) beads were used in a 0.9:1 ratio to purify the DNA fragments and remove primers. The concentration of each of the ten pools was established using Qubit high‐sensitivity dsDNA assay (ThermoFisher Scientific, Paisley, UK) and informed the volumes of each pool to be combined to compile three final pools in which samples were approximately equimolar. Because of the large number of COI samples exceeding the number of unique tagged primer combinations, two COI pools were compiled for each plate, each containing the same tagged primer combinations. The NEXTflex Rapid DNA Sequence Kit (Bioo Scientific) was used to prepare the samples by blunt‐end ligation (Figure S1b) and PCR amplification of libraries to integrate adapters for Illumina sequencing. This manufacturer's protocol for library preparation without size selection was followed, and final libraries were analyzed using a TapeStation 2200 to confirm successful addition of adapters. The COI libraries were loaded onto an Illumina MiSeq for sequencing with a 500‐cycle V2 cartridge, and a 500‐cycle Nano cartridge was used for ITS2.

### 2.5 Bioinformatics Processing

Bioinformatic analysis followed Drake et al. (2022). The process was carried out for each of the three sequencing libraries separately. First, the rate of MID‐tag primers truncation was calculated, which was deemed acceptable (mean 9%). FastP (Chen et al. 2018) was then used to trim adapters, pair forward and reverse reads, and remove low‐quality reads (minimum sequence length of 125 bp and quality threshold of Q33). After this, there were 6,486,267 reads in COI index 1, 6,730,882 in COI index 2, and 772,865 in ITS2 (includes full‐grown and nestling samples). Mothur (Schloss et al. 2009) was used to assign the sequences to their respective sample according to their unique tag combination and trim primer sequences (allowing one mismatch). Reads were then demultiplexed to obtain one file of prey sequences per unique tag combination (i.e., per great tit sample).

The ‘unoise3’ command in Usearch (Edgar 2010) was used to identify and correct reads with sequencing errors, remove chimeras and assign sequences to zOTUs (zero‐radius operational taxonomic units; Edgar and Flyvbjerg 2015; Edgar 2016). A closest species match was determined via the ‘blastn’ command in BLAST+ (Basic Local Alignment Search Tool, Camacho et al. 2009) by matching sequences from GenBank using 97% similarity.

Only the top hit for each zOTU was retained, using *dplyr* in R (v0.7.8; Wickham et al. 2019), based on bit‐score. MEGAN6 Community Edition (MEtaGenome Analyser, v6.15.2, Huson et al. 2016) was used to assign taxon names to zOTUs. ZOTUs corresponding to the same taxon were aggregated. Taxa with fewer than ten reads attributed to a given sample were removed from that sample as likely errors. The maximum number of reads for each taxon present across negative controls or unused MID‐tag combinations was subtracted from all other read counts for that taxon to remove potential contamination (Drake et al. 2022). Read counts were converted to binary presence/absence data given the quantitative biases associated with metabarcoding (Yu et al. 2012; Clare et al. 2014; Deagle et al. 2019). At this point, the two separate COI libraries were aggregated. Non‐target detections, such as feather mites, non‐European species, marine species, water moulds, amoeba, and positive control taxa, were removed, alongside known lab contaminants (German cockroach, *Blattella germanica* ).

All analyses were performed at the species level. When there was no species level data for an identified genus, the genus data was included as ‘*Genus* sp.*’*, e.g., *Fagus* sp. For analysis, any taxa at family level or above, and any sample that did not contain dietary data at species level were removed. Additionally, two plant genera, *Citrus* (citrus fruits) and *Cucumis* (cucumbers and melons) were removed since they were likely derived from private gardens and are unlikely to be primary food sources.

### 2.6 Statistical Analysis

#### 2.6.1 Sampling Completeness

Sampling completeness analysis was conducted in R v4.0.3 (R Core Team 2019). Analyses were carried out at the site level using the full datasets of animal prey and plant detections, excluding duplicate samples from birds sampled multiple times. To assess sample coverage, coverage‐based rarefaction and extrapolation were carried out separately for the COI and ITS2 datasets using the ‘iNEXT’ package v3.0.1 with species represented by frequency‐of‐occurrence across samples (Chao et al. 2014; Hsieh et al. 2016). A plot of cumulative sample coverage per detection was generated via iNEXT for each dataset. Sample coverage was also assessed and plotted separately for the different habitats, seasons, sexes and ages compared to confirm equivalent completeness of sampling.

#### 2.6.2 Diet Composition Comparison

R v4.1.0 (RStudio Team 2019; R Core Team 2021) was used for all dietary analyses. To investigate individual differences in dietary composition of the species community as a whole, we used the package *mvabund* v4.1.12 (Wang et al. 2012) to generate multivariate generalised linear models (manyglm) with a binomial error family and ‘cloglog’ link function. Global model results were tested by likelihood ratio test using the ‘anova’ function with Monte Carlo (parametric bootstrap) resampling as recommended in Wang et al. (2012) for presence/absence data. The *p.uni = adjusted* argument was used to investigate univariate effects (Wang et al. 2012). Model fit was checked by plotting the model residuals. Random effects cannot be incorporated into a manyglm, so duplicate samples from the same bird in the same season in each dataset were randomly removed. Dietary differences within and between groups were visualised via NMDS (nonmetric multidimensional scaling) plots using the ‘metaMDS’ function in the *vegan* package v2.5.7 (Oksanen et al. 2020) with Jaccard dissimilarity.

#### 2.6.3 Invertebrate and Plant Richness and Dietary Composition

(i) Richness: We ran analyses to investigate individual dietary richness using two separate datasets. In the first, we examined the invertebrate diet (plants excluded) for the combined seasons: spring (two years: May to June in 2017 and 2018) and winter (single year: November 2017 to February 2018). Two generalized linear mixed models (GLMMs) were used to avoid having high collinearity when including all five interactions in a single model. Both models had a Poisson distribution and a log link function, and the response variable was the total number of invertebrate species consumed. The first model included habitat × season and year only. The second model included the four interactions: age × season, age × habitat, sex × season, sex × habitat, and year, but not habitat × season. Both models included study site and bird ID as random effects.

In the second analysis on dietary richness, we examined the winter diet alone so we could investigate the full diet in winter that included both plants and invertebrates. The response variable was the number of winter plant and invertebrate species consumed and the model was a GLMM with a negative binomial distribution. The model included habitat, age, sex, and age × sex as an interaction. Study site was included as a random effect. Interactions with habitat could not be included due to small sample sizes. Two packages *Dharma* v0.4.1 (Hartig 2021) and *Performance* v0.9.1 (Lüdecke et al. 2021) were used to check model fit and test for overdispersion for all richness models, and the package *emmeans* v1.7.4.1 was used to examine significant interactions (Lenth 2022).

(ii) Composition: For dietary composition, we first used a multivariate generalized linear model (manyglm) to examine the main effect of season on invertebrate prey community composition. This model included season as well as habitat (coniferous or mixed‐deciduous), age (adult: > 1 year old; or first year: < 1 year old) and sex as main effects. We next investigated the two seasons separately because, first, the large differences in diet between the seasons inflated the number of species × season combinations with zero entries, and second, because of the need to avoid three‐way interactions involving season. Therefore, we determined invertebrate dietary composition in the spring only with a second model including the main effects of age, sex, year (2017 or 2018) and habitat, with the addition of age × sex, age × habitat and sex × habitat as interactions. Finally, we investigated the winter diet (plant and invertebrate) composition using a multivariate generalized linear model that included age, sex and habitat as main effects and age × sex as an interaction.

Some species were rare and present in a small number of diet samples. To explore how big a role rare species played in the diet composition, all composition models were run both with the full number of species and then with only the species that were present in more than an arbitrary 10% of samples. For all models, the results of the main effects were taken from a main effects model only, and the results from the interactions were taken from the full model. Study site was not included in the composition models because a manyglm cannot incorporate random effects which meant that, as some of the sample sizes were very low, we needed to combine samples from different sites in order to look at habitat effects. Results of *p* < 0.1 are discussed as non‐significant trends (Muff et al. 2022).

## 3 Results

### 3.1 Sequencing Output and Sampling Completeness

After initial processing, invertebrates were identified in 122 spring and 66 winter faecal samples. Of the 436 invertebrate taxa identified using COI, 256 were identified to species or subspecies level, and the remaining taxa were assigned to coarser taxonomic levels. After removal of likely false positives, non‐European, and non‐diet species, 228 invertebrate species/subspecies remained in the dataset (Table S2), of which 160 invertebrate species from 54 families were used in the analysis, since the remainder were present in nestling samples and samples from urban study sites, which were not used for this paper.

Of 85 plant DNA sequences identified in the winter samples using ITS2, 45 were identified to species and subspecies level, and the remaining sequences were assigned to coarser taxonomic levels. The final winter dataset included 47 plant species (including two *Genus* sp.; Table S3) and 80 invertebrate species. The plant and invertebrate data for each winter sample was combined into the same dataset and, after processing the data, we had 109 samples with invertebrate or plant material, or both, present (see Table S4).

Sampling achieved a high degree of coverage in both the COI (estimated coverage = 98.0%, Figure S2a) and ITS2 (95.5%, Figure S2b) datasets. Sampling was also relatively complete across each category compared (deciduous = 97.1%, coniferous = 85.8%, winter = 90.9%, spring = 97.6%, adult = 95.1%, first year = 94.4%, female = 94.2%, male = 94.1%; Figure S3).

### 3.2 General Diet Description

A total of 160 invertebrate species in 141 genera of 22 guilds were identified across years and seasons (Tables 1 and S2). In spring, 132 invertebrate species of 20 guilds were identified, and in winter, 80 invertebrate species of 17 guilds were identified. Moths, spiders (not including Clubionidae), hoverflies, parasitoid wasps, and aphids were the top five most prevalent guilds within samples in spring. In winter, the top five were moths, spiders, true flies, gall wasps, and sac spiders (Clubionidae). Also in winter, 47 plant species of 20 orders were identified in 106 samples taken from both habitats (Table S3). Four species were present in over 39% of samples, a further 10 in more than 5%, and the rest were in less than 5%. The most common natural species were beech (*Fagus* sp.; 89%) and *Rubus* sp. (fruits such as blackberry and raspberry; 45%). Species provided as bait when trapping the great tits were also moderate‐highly prevalent (*Helianthus* sp., sunflower, 75%; *Arachis* sp., peanut, 39%).

**TABLE 1:** Number and percentage of samples, across both years, that had at least one species of each group of invertebrates, for all 188 samples that had invertebrate material present, and split by season (N: Spring = 122, winter = 66).

*Invertebrates*	Group			Both seasons		Spring		Winter	
		No. of species	No. of families	No. of samples	%	No. of samples	%	No. of samples	%
	Moths	70	14	166	88	122	100	44	67
	Spiders	15	8	87	46	55	45	32	48
	Hoverflies	8	1	77	41	68	56	9	14
	Parasitoid wasps	22	2	71	38	56	46	15	23
	Aphids	8	1	55	29	50	41	5	8
	Sac spiders	4	1	41	22	24	20	17	26
	True flies	6	6	37	20	18	15	19	29
	Weevils	3	1	28	15	20	16	8	12
	Moth flies	2	1	26	14	16	13	10	15
	Shield bugs	1	1	21	11	6	5	15	23
	Crane flies	1	1	18	10	15	12	3	5
	Beetles	6	5	17	9	11	9	6	9
	Gall wasps	1	1	17	9	0	0	17	26
	Midges	1	1	15	8	15	12	0	0
	Butterflies	3	2	9	5	9	7	0	0
	Gnats	1	1	9	5	1	1	8	12
	Lacewings	2	2	8	4	4	3	4	6
	Bees	2	1	7	4	7	6	0	0
	Mayflies	1	1	6	3	4	3	2	3
	Slugs	1	1	5	3	5	4	0	0
	Sawflies	1	1	4	2	4	3	0	0
	Earwigs	1	1	1	1	0	0	1	2

*Note:* In total, 160 invertebrate species of 141 genera were identified and present in the dataset. Groups are ordered according to the percentage of samples for both seasons. See Table S3 for full list of invertebrate prey species in the diet. Note that ‘sac spiders’ are from the family Clubionidae only and ‘spiders’ include all other families.

### 3.3 Dietary Species Richness

**FIGURE 2:**
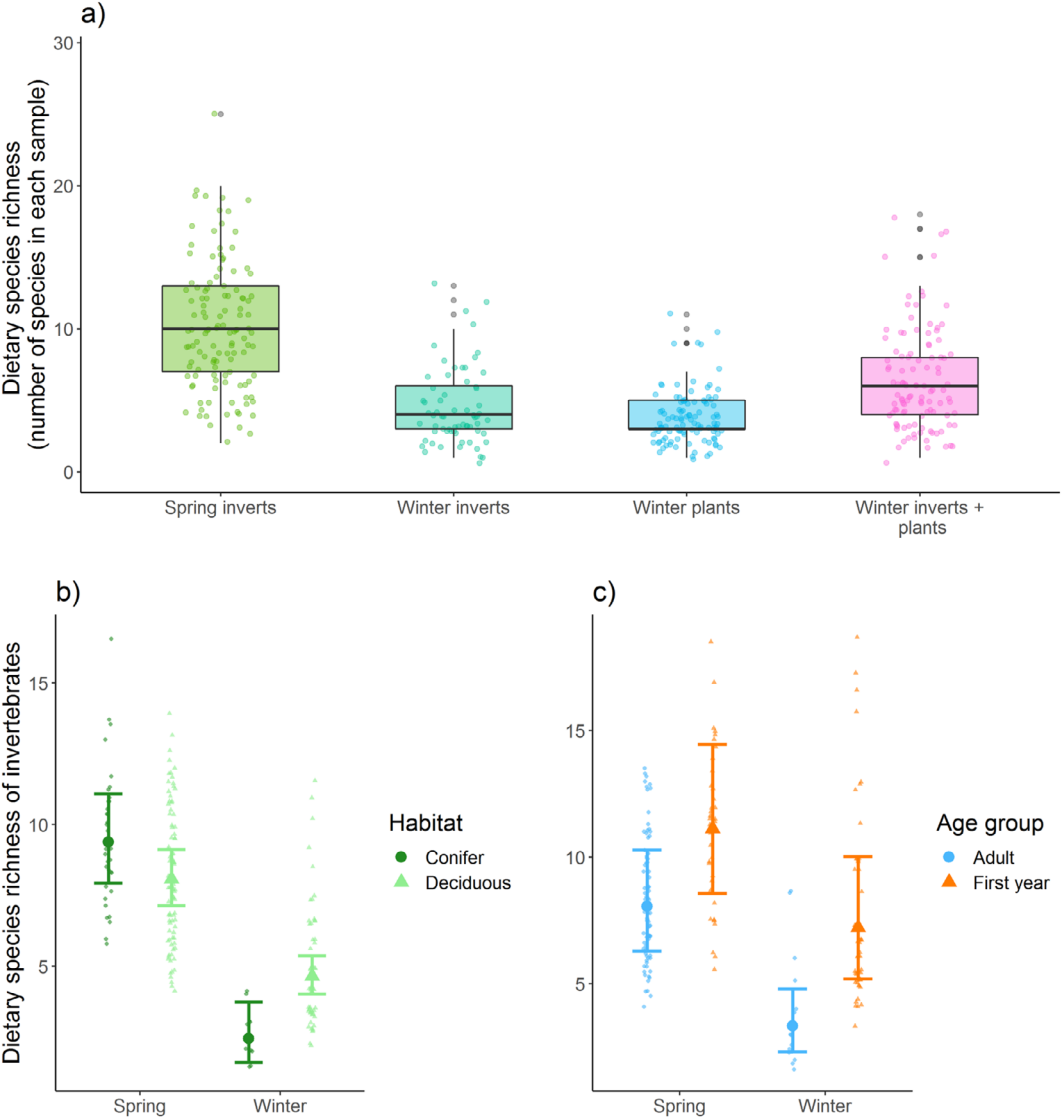
Dietary species richness (number of species in each individual sample) for (a) spring invertebrates (*N* = 122 samples), winter invertebrates (*N* = 66), winter plants (*N* = 106) and the combined winter diet of invertebrates and plants (*N* = 109). Boxes show the median and interquartile range with whiskers extending to 1.5 × IQR above and below. Individual dietary species richness of invertebrates for seasons (spring and winter) and (b) habitats (conifer and deciduous) and (c) age groups (adults and first years). For (b) and (c), partial residuals from the GLMM are shown.

The mean number of invertebrate species present in any one sample was 8.2 ± 4.72 SD (range 1–25; Figure 2a). Due to model constraints (see Materials and Methods), we ran two separate models for dietary invertebrate richness throughout the year (Table 2a,b). The first model showed that richness varied among years and that great tits consumed more invertebrate species in spring 2018 than in 2017 (Table 2a). It also showed that great tits consumed fewer invertebrate species in the winter than in the spring, as expected, but that this difference was less pronounced in samples from mixed‐deciduous woodlands than those from coniferous (habitat × season, Table 2a; Figure 2b). In winter, more invertebrate species were eaten in mixed‐deciduous compared to those in coniferous habitats, but in spring there was no difference between habitats (Emmeans: spring: Est = 0.15, SE = 0.09, *Z* = 1.68, *p* = 0.09; winter: Est = −0.64, SE = 0.22, *Z* = −2.85, *p* = 0.004; Figure 2b).

**TABLE 2:** Species richness GLMMs for invertebrates all year round: (a) including just one interaction and (b) including four interactions (model would not run with 5 interactions). (c) is a species richness model for invertebrates and plants but only in winter.

Model	Variable	Estimate	SE	*Z* value	*p* value	*N*
(a) Invertebrates only all year (with one interaction)	Intercept	2.24	0.08	26.44	**< 0.001**	188
	Habitat (conifer)	−0.15	0.09	−1.68	0.09	
	Season (spring)	−1.34	0.22	−5.97	**< 0.001**	
	Year (2017)	0.27	0.07	4.00	**< 0.001**	
	Habitat × Season	0.79	0.24	3.33	**< 0.001**	
(b) Invertebrates only all year (with four interactions)	Intercept	2.09	0.12	16.8	**< 0.001**	
	Age (adult)	0.33	0.15	2.18	**0.03**	
	Habitat (conifer)	−0.08	0.13	−0.63	0.53	
	Season (spring)	−0.88	0.19	−4.72	**< 0.001**	
	Sex (female)	−0.04	0.14	−0.30	0.77	
	Year (2017)	0.27	0.07	3.90	**< 0.001**	
	Age × Sex	0.02	0.14	0.15	0.88	
	Age × Habitat	−0.30	0.16	−1.91	0.06	
	Age × Season	0.45	0.20	2.24	**0.03**	
	Sex × Habitat	0.21	0.15	1.40	0.16	
	Sex × Season	−0.31	0.16	−1.85	0.06	
(c) Winter only: invertebrates and plants	Intercept	1.64	0.19	8.84	**< 0.001**	109
	Age (adult)	0.24	0.23	1.04	0.30	
	Habitat (conifer)	0.07	0.21	0.35	0.73	
	Sex (female)	−0.07	0.24	−0.31	0.76	
	Age × Sex	−0.04	0.27	−0.14	0.88	

*Note:* Reference level for main effects is in brackets. Site and bird were random effects in all models. *p* values greater than 0.05 are shown in bold.

In the second model of invertebrate dietary richness throughout the year, there was some evidence for dietary richness being higher for first‐year birds than adults (main effect, Table 2b). This difference was most pronounced in winter (age × season: Table 2b; Figure 2c), and there was also a tendency for the age difference to be dependent on habitat type (age × habitat, Table 2b). There was less evidence for pronounced effects of sex. Specifically, there was no main effect, no interaction with age, and weak tendencies for effects of sex to be dependent on season (sex × season: Table 2b).

In winter, for which we had both invertebrates and plants, there was no evidence for any effects of habitat, age, or sex on dietary species richness (Table 2c).

### 3.4 Invertebrate Composition Throughout the Year

**FIGURE 3:**
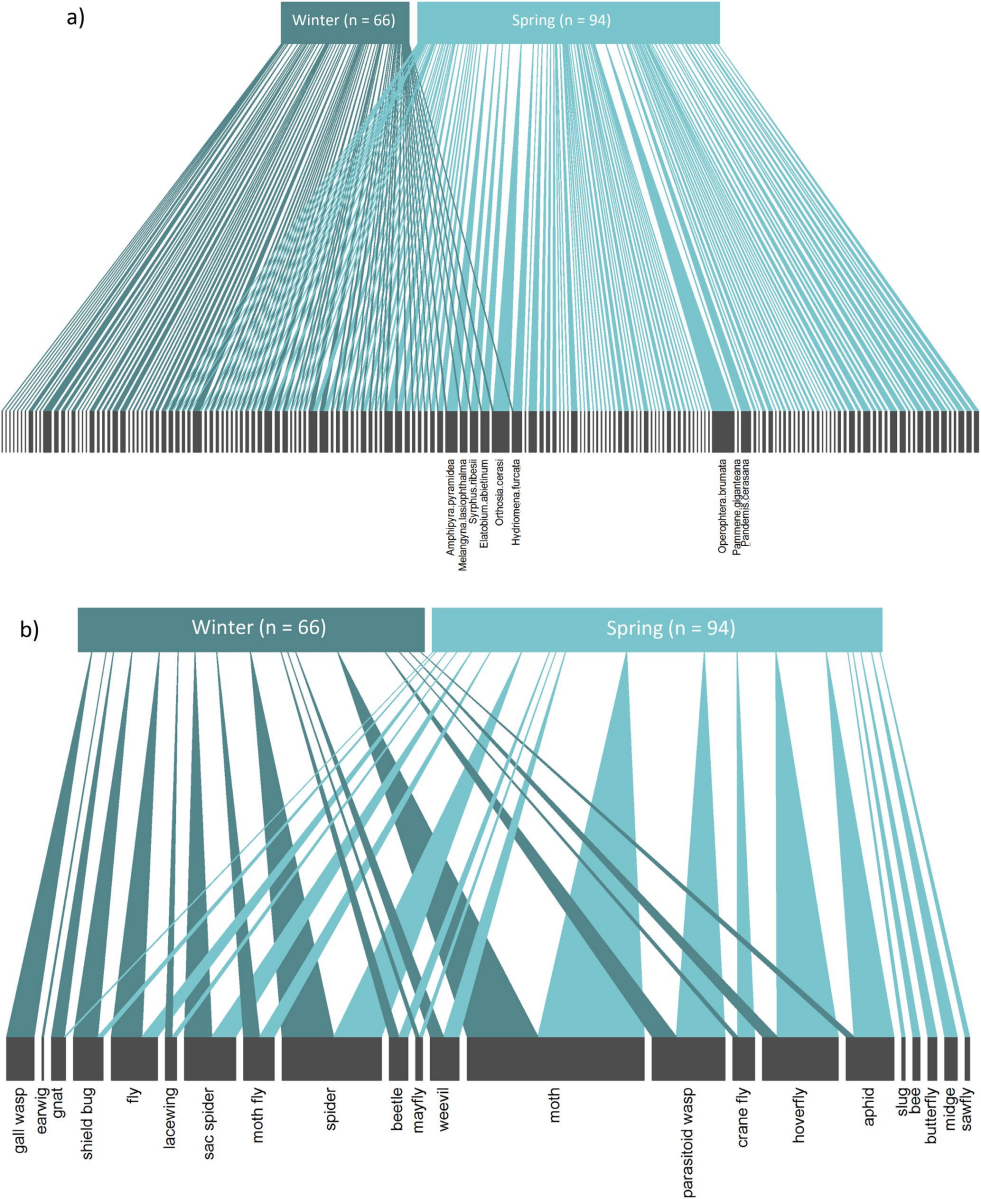
The (a) individual invertebrate species and (b) invertebrates grouped into guilds, consumed in the spring (*n* = 94) and winter (*n* = 66). The width of the links represents the number of birds from each season that consumed each prey species or guild (the lower bars). Invertebrate species or guilds that were present in the diet of more individual birds have a wider bar. For (a) the most prevalent species are labelled. All bipartite plots were created using the *bipartite* package (v2.17).

Invertebrate prey composition differed between spring and winter (Table 3a; Figure 3). Prey guilds that were in a higher percentage of samples in winter than in spring included spiders, flies (true flies, moth flies and gnats), shield bugs, gall wasps, lacewings, and earwigs (Table 1; Figure 3b). Although the five most prevalent species in spring were all moths, in winter the six most prevalent species included a gall wasp ( *Neuroterus quercusbaccarum* : 26% of winter samples, 0% in spring), shield bug ( *Acanthosoma haemorrhoidale* : 23% cf. 5% in spring), moth fly, and three spider species (Table S2). Univariate tests from the multivariate generalized linear model analysis showed that there was a significant difference in the prevalence of 37 species between spring and winter: 19 were moths and the rest were flies, hoverflies and midges, parasitoid wasps, spiders, an aphid, and a gall wasp species (Table S5a). Of these, the most prevalent in spring was the winter moth, *Operophtera brumata* (present in 71% of samples from the spring dataset), which was entirely absent from the winter diet (prevalence of all individual species for the full dataset is shown in Table S2). The Noctuidae moth *Orthosia cerasi* was also prevalent in more than half of spring samples (54%), but in only 3% of winter samples. Age, sex, and habitat all significantly related to dietary‐prey community composition in this model, which is explored in more detail within seasons below.

**TABLE 3:** Multivariate generalised linear models of species composition in the diet of great tits at different times of the year and with different datasets.

Data	Variable	LRT	*p*	*N*	*N* sub‐groups	
(a) Invertebrates all year	Season Habitat Sex Age	969.1 329.6 303.9 195.9	**0.001** **0.001** **0.001** 0.042	160	Spring: 94 Conifer: 34 Female: 81 Adult: 79	Winter: 66 Deciduous: 126 Male: 79 First year: 81
(b) Invertebrates in spring	Habitat Year Sex Age Age × Sex Age × Habitat Sex × Habitat	319.3 395.1 257.6 168.1 129.2 87.7 81.7	**0.001** **0.001** **0.001** 0.06 **0.001** **0.03** 0.07	101	Conifer: 27 2017: 42 Female: 46 Adult: 66	Deciduous: 74 2018: 59 Male: 55 First year: 35
(c) Invertebrates in spring (excluding species in < 10% of samples)	Habitat Year Sex Age Age × Sex Age × Habitat Sex × Habitat	163.1 168.9 95.2 31.1 49.1 40.4 36.9	**0.001** **0.001** **0.001** 0.77 0.18 0.63 0.65	100	Conifer: 26 2017: 42 Female: 46 Adult: 66	Deciduous: 74 2018: 58 Male: 54 First year: 34
(d) Plants and invertebrates in winter	Habitat Sex Age Age × Sex	106.8 173.3 118.9 309.3	**0.05** **0.006** 0.06 **0.001**	109	Conifer: 20 Female: 61 Adult: 25	Deciduous: 89 Male: 48 First year: 84
(e) Plants and invertebrates in winter (excluding species in < 10% samples)	Habitat Sex Age Age × Sex	23.8 8.30 14.0 13.7	**0.002** 0.26 0.06 0.44	105	Conifer: 17 Female: 58 Adult: 22	Deciduous: 88 Male: 47 First year: 83

*Note:* Models a–c are invertebrates only; models d–e additionally include plants. Model a includes data for the whole year, models b–c are for spring, and d–e are for winter. Models a, b, and d include all species, while c and e exclude species that occur in less than 10% of their respective datasets. LRT is the likelihood ratio test. All the LRT and *p* values come from the ANOVA comparison between the reduced model (without the variable of interest) and the full model. The values for all main effects come from a model with the main effects only (i.e., no interactions included). The values for the interactions come from the full model with all main effects and interactions included. Sample size for the whole dataset and sample sizes for individual sub‐groups are shown. We did not test for age × habitat and sex × habitat interactions in the winter dataset due to low sample size. *p* values greater than 0.05 are shown in bold.

### 3.5 Invertebrate Composition in Spring

**FIGURE 4:**
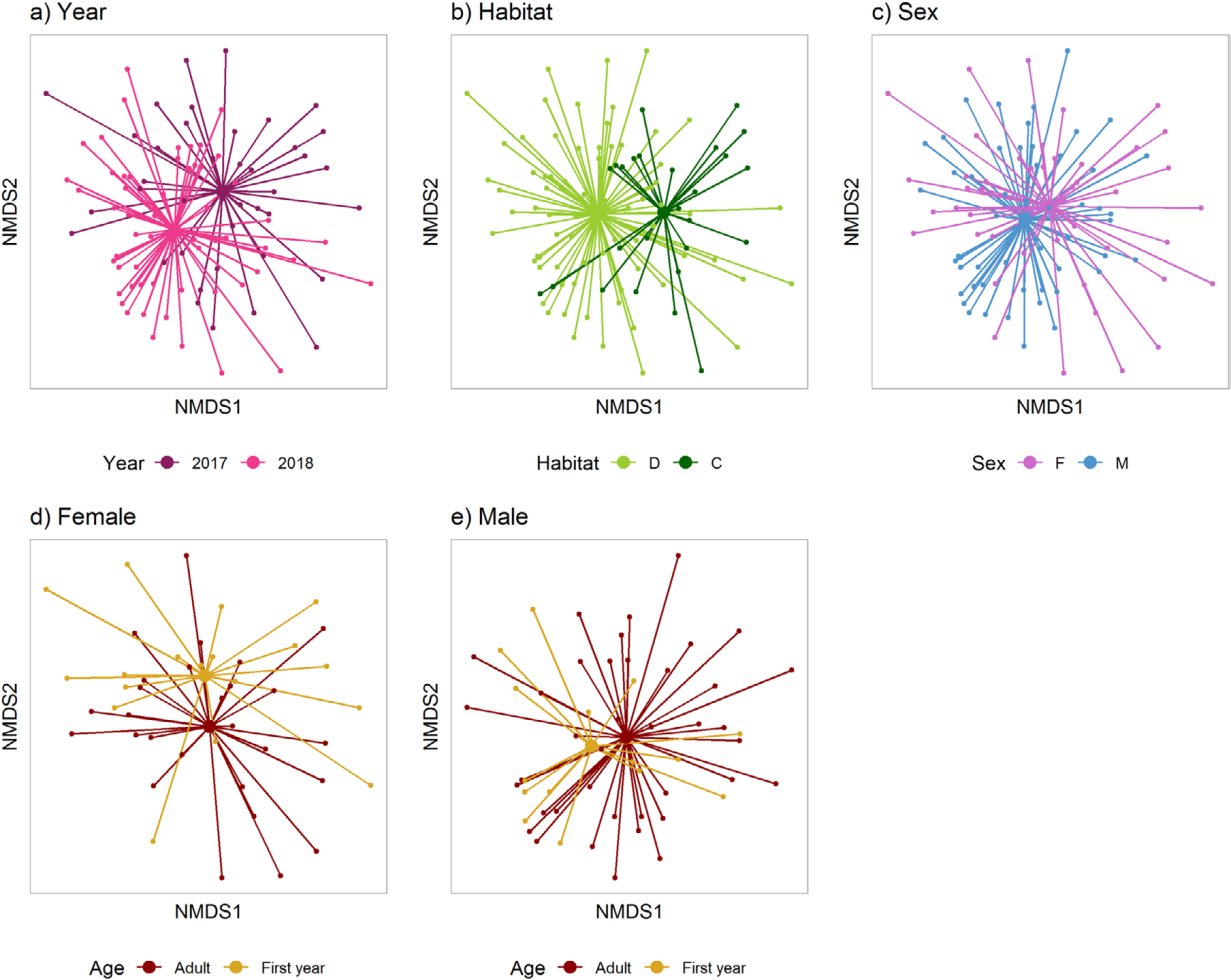
Non‐metric multidimensional scaling (NMDS) plots show differences in spring invertebrate diet among: (a) years (41 in 2017, 58 in 2018), (b) habitats (73 D = deciduous, 26 C = conifer), (c) sexes (45 F = females, 54 M = males, (d) ages, for females (27 adults, 18 first years) and (e) ages, for males (39 adults and 15 first years). Each point shows an individual and the connecting lines join the individual to the mean of its year, habitat type, sex class or age/sex class. The Jaccard index was used to calculate the distances between samples. Data includes no duplicates from the same individual. Two outliers were removed (females). The corresponding multivariate generalised linear model analysis is given in Table 3b.

Spring diet composition varied with all of the main effects (Table 3b). First, it varied among years (Table 3b; Figure 4a). There was no evidence that any of the top seven most prevalent invertebrates varied among years (Tables S2 and S5b), but univariate analyses identified six other species that differed in prevalence among years ( *Syrphus ribesii* 24% prevalence from spring dataset*; Clubiona reclusa* 20%*; Meliscaeva auricollis* 17%*; Syrphus torvus*, 12%*; Enytus montanus*, 11%; *Glyptapanteles porthetriae* , 11%; Table S5b). Second, spring diet composition varied between habitats (Table 3b, Figure 4b); univariate analysis suggested that five species varied between habitats, including two of the top four most prevalent species: (*Orthosia cerasi*, 65% Deciduous (D), 26% Conifer (C); *Hydriomena furcata* , 22% D, 59% C; *Agrochola lota* , 7% D, 48% C; *Elatobium abietinum*, 15% D, 52% C; *Meliscaeva auricollis*, 8% D, 41% C; Table S5b).

Third, spring invertebrate species composition differed between the sexes (Table 3b; Figure 4c) and univariate tests provided evidence for differences at the species level in two parasitoid wasps and one moth fly (Tables S3 and S5b). Fourth, there was a tendency for a main effect of age, and there was a significant interaction between age and sex (Figure 4d,e; Table 3b), but no univariate tests were significant for either of these effects, and there was weak support for interactions with habitat for age and sex (Table 3b).

Finally, the analysis on the dataset that excluded species in < 10% of samples (Table 3c) retained the main effects of habitat, year, and sex, and there were no significant differences between the datasets for the univariate effects on individual species. The analysis on the reduced dataset did differ from the full dataset for age in that it provided no support, either as a main effect or in any interaction. There was also no evidence for the interaction between sex and habitat that had a tendency towards significance in the full dataset.

### 3.6 Invertebrates and Plant Dietary Composition in Winter

**FIGURE 5:**
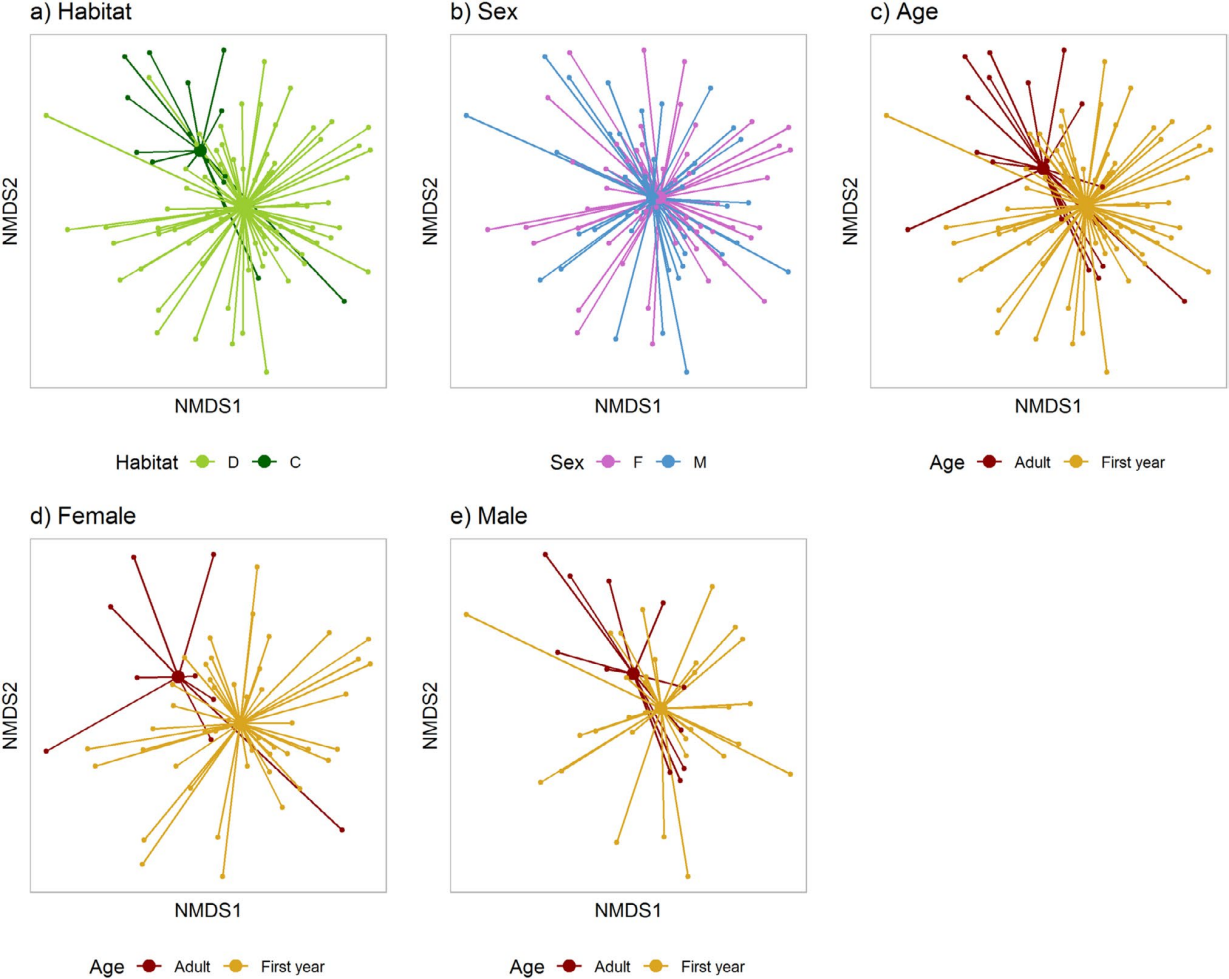
Non‐metric multidimensional scaling (NMDS) plots show differences in plant and invertebrate winter diet among: (a) habitats (87 D = deciduous, 17 C = conifer), (b) sexes (46 M = males, 58 F = females), (c) ages (21 adults, 83 first years), (d) ages, for females (9 adults, 49 first years), and (e) ages, for males (12 adults and 34 first years). Each point shows an individual and the connecting lines join the individual to the mean of its habitat type, sex or age class. The Jaccard index was used to calculate the distances between samples. Data includes no duplicates from the same individual. Five outliers were removed (four adults, one first year and three females, two males). The corresponding multivariate generalised linear model analysis is given in Table 3d.

**FIGURE 6:**
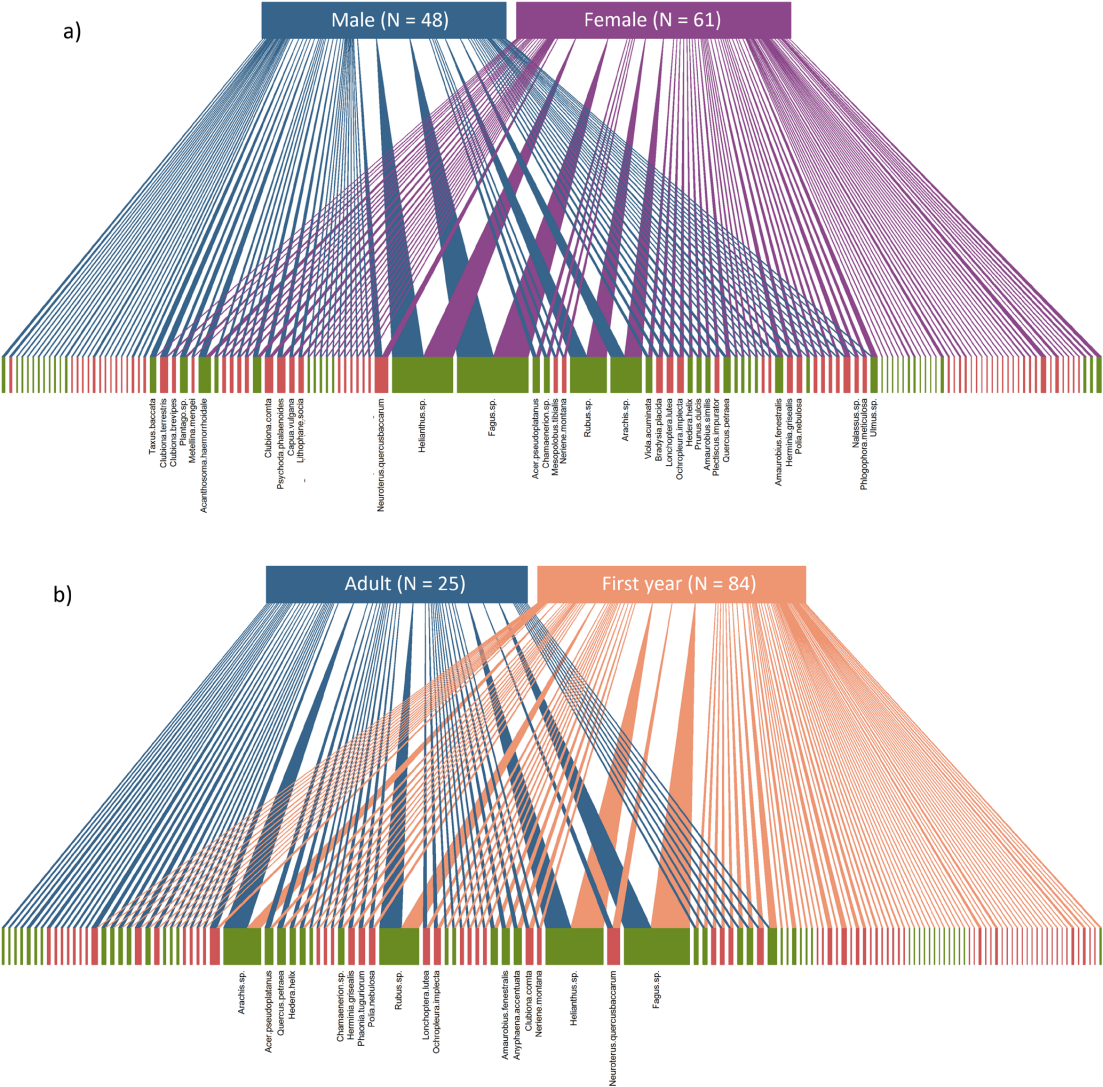
The invertebrate (red lower bars) and plant species (green lower bars) consumed in the winter diet of (a) male (*N* = 48) and female (*N* = 61) and (b) adult (*N* = 25) and first year (*N* = 84) great tits. The width of the links represents the number of birds from each group that consumed each prey species (the lower bars). Invertebrate species that were present in the diet of more individual birds have a wider bar. The most prevalent species are labelled. All bipartite plots were created using the *bipartite* package (v2.17).

In winter, the top four most prevalent species were all plants (42%–94% prevalence; Table S4). Eleven further species occurred in 10% or more of the samples, and four of these were also plants. Composition varied with habitat (Table 3d; Figure 5a); univariate tests identified that beech (*Fagus* sp.) prevalence differed among habitats, with 93% and 55% being consumed in mixed‐deciduous and conifer, respectively (Table S5d). Composition also differed between the sexes (Tables 3d, S4; Figures 5b, 6a) though no univariate tests were significant. There was weak support for a difference between adults and first‐years (Table 3d; Figures 5c, 6b). Specifically, univariate tests suggested a difference in beech (*Fagus* sp.), which occurred in 96% of first‐years (*n* = 81) and 52% of adults (*n* = 25; Tables S4 and S5). The interaction between age and sex (Table 3d; Figure 5d,e) was also significant, though the only significant individual species in univariate tests was the fly *Phaonia tuguriorum* (8% winter prevalence, 18% adult female, 10% first‐year female, 0% all males; Table S4).

When excluding species that occurred in < 10% of samples, age still had a tendency towards significance with a significant univariate effect of *Fagus* sp. (Table S5e). Habitat remained a significant main effect and had two significant species in the univariate analyses (*Fagus* sp. and peanut *Arachis* sp. with the gall wasp *Neuroterus quercusbaccarum*, having a tendency towards significance; Table S5e). In the reduced dataset, the sex main effect and the age × sex interaction were no longer significant (Table 3e).

## 4 Discussion

Most studies on the diet of tit species and passerine species focus on nestlings, especially in the context of phenology (Nour et al. 1998; Naef‐Daenzer et al. 2000; Wilkin et al. 2009; Pagani‐Núñez et al. 2011, 2017; Höhn et al. 2024). Birds are typically harder to study full‐grown (post‐fledging) due to their cryptic foraging habits and the small size of their prey, especially in the non‐breeding season (Gibb 1954; Betts 1955; Vel'ký et al. 2011). We have, for the first time to our knowledge, examined the diet of adult and first‐year great tits using DNA metabarcoding. Sampling had high completeness and showed a high degree of coverage, indicating the likely robustness of our results.

### 4.1 General Patterns in Diet

DNA metabarcoding detected 160 invertebrate species from 160 samples that were used in the analysis, across the seasons and years. Compared to an estimated 432 prey MOTUs (molecular operational taxonomic unit) recorded from 772 faecal samples in the blue tit in Scotland (Shutt et al. 2020), our invertebrate diversity is relatively small. This may be because of our lower number of samples, the fragmented nature of our study area (O'Shea et al. 2018), and the generally low diversity in Ireland compared to Britain and continental Europe. Alternatively, differences in temporal and spatial sampling could explain variation between our study and that of Shutt et al. (2020). Compared to the landmark study on great tits by Betts (1955), we detected the same six main invertebrate orders (Lepidoptera, Hymenoptera, Coleoptera, Hemiptera, Diptera, Araneae), but we also detected four previously unreported orders (Ephemeroptera, Neuroptera, Demaptera and Pulmonata). Similarly, 20 species of Noctuidae and 15 species each of Geometridae and Tortricidae were detected, aligning with other studies indicating that moths are an important component of tit diets (Betts 1955; Royama 1970; van Balen 1973; Cramp and Perrins 1993). We also identified 10 species of Erebidae moths that were not present in the other studies mentioned. Although some of the new species we detected could be explained by geographical or study site differences, our use of DNA metabarcoding may explain the difference because, for example, non‐genetic methods are unable to identify soft‐bodied taxa such as Pulmonata (Moran et al. 2019; Ruppert et al. 2019).

Most dietary taxa were rare with 113 of 160 invertebrate taxa occurring in ≤ 5% of samples, which aligns with similar metabarcoding studies on the blue tit (Shutt et al. 2020) and on other insectivorous passerines (Garfinkel et al. 2022; Bookwalter et al. 2023). Collectively, these rare dietary detections formed a large component of the diet (30% of prey item detections across all samples; *n* = 511 of 1500; from Table S2), and totaled twice as many detections as the top four most prevalent species in the diet (*n* = 230). Although it should be unsurprising that rare species can be an important component of diet in a generalist species, these results are a rare demonstration of this principle among full‐grown birds in an omnivorous species, made possible by DNA metabarcoding.

Our study is consistent with others that found Lepidoptera to be the dominant order consumed by great tits (Betts 1955; Royama 1970; van Balen 1973; Barba et al. 1996). As in many previous studies of great tit diet (Gibb 1954; Betts 1955; van Balen 1973; van Noordwijk et al. 1995; Buse et al. 1999), the winter moth *O. brumata* was the most abundant invertebrate in our spring samples (71%). However, the common quaker moth *Orthosia cerasi*, a species not recorded by Betts (1955), was almost as prevalent (54%) as the winter moth. Similarly, the prevalence of *O. cerasi* in the diet of Irish great tits is far higher than the 20% prevalence found in the diet of hawfinch *Coccothraustes coccothraustes* in Europe (Stenhouse et al. 2023), and the < 1% prevalence found in blue tits (Shutt et al. 2020). Furthermore, the summed prevalence of the 10 most prevalent moth species excluding the winter moth in our study is 338, four times more than the winter moth alone. Much research has focused on how climate change can lead to potential mismatches between the emergence of winter moth larvae and the timing of breeding in European tit species (Visser and Holleman 2001; Vedder et al. 2013; Simmonds et al. 2020). Less is known about the phenology of other European Lepidopteran species, although in North America there is substantial among‐species variation in phenology in *Quercus* spp. woodlands (Forkner et al. 2008). Thus, our demonstration that great tits consume a variety of Lepidopteran species raises the question of whether these prey items differ in their phenology in our woodland system and, if so, whether this could affect how populations respond to climate change.

Plant material was the most prevalent food eaten in winter, aligning with Vel'ký et al. (2011), the only other study that details the winter diet of great tits. Beechmast (*Fagus*) was the most prevalent dietary plant item in winter (89%) and was even more dominant than the food provided at feeders. Beechmast is widely known to be an important winter food source (Gibb 1954; Betts 1955; van Balen 1980; Perdeck et al. 2000) and can be a major driver of annual survival and population fluctuation (Perrins 1966; van Balen 1980; Gosler 1987; Perdeck et al. 2000). However, *Rubus* species were found in 45% of samples, 10 other plant species were detected in 5% or more samples, and a further 33 species of plant were detected in at least one sample, suggesting that, collectively, other plant species are likely just as important.

The freely and consistently available plant‐based food provided at feeders during the winter was also highly prevalent (75% for sunflower seeds; 39% for peanuts). Nevertheless, natural plant species remained similarly prevalent in the diet (Tables S3 and S4), as indeed did invertebrates. There is some evidence to suggest that supplemental food in winter can impact overwinter survival (van Balen 1980; Kallander 1981; Brittingham and Temple 1988, but see Plummer et al. 2013, 2018 for evidence of supplementary feeding reducing breeding productivity) and yet it appears insufficient as a food source on its own. Possible reasons for this include supplemental food being of limited nutritional value, or increased predation risk and competition at feeders. Our data on plants are rare among similar studies because although plants are clearly the focus in studies on frugivores (e.g., Herrera 1984), or on seed dispersal (Jordano 1982), plant materials are generally overlooked in ornithological diet studies of generalist species.

### 4.2 Temporal Variation

The bipartite network showed a larger number of invertebrate species in spring than in winter. There was some overlap between the seasons, and while many more invertebrate species were present only in spring, some species were unique to the winter diet. These results are most likely explained by the greater activity of invertebrates during the spring, but also by temporal differences in the life cycles of different species (Aitchison 1984; Eitzinger and Traugott 2011). Invertebrate dietary composition and richness differed between years, likely reflecting annual differences in invertebrate populations due to, for example, climatic conditions (Bozinovic et al. 2011; Barnett and Facey 2016). Annual variation in invertebrate‐guild dietary richness has also been reported in great tit nestlings using video recordings (Olivé‐Muñiz et al. 2021; Sinkovics et al. 2021). The higher richness we found in 2018 may indicate a lower abundance of preferred prey, so great tits had to diversify their diet to include other species. Alternatively, 2018 could have had more favorable weather conditions, leading to generally higher invertebrate abundance and diversity compared to 2017. Whether and why the diet of passerine species varies annually is poorly understood (see Wiens and Rotenberry 1979; Durst et al. 2008 for riparian and shrubsteppe species) but our results show that DNA metabarcoding may provide useful insight.

### 4.3 Habitat Variation

There were differences in the composition of invertebrate prey in the diet of great tits from coniferous and mixed‐deciduous woodlands, in both spring and winter. This is to be expected since invertebrate communities differ in these habitats (Ozanne 1999; Humphrey et al. 2002; Finch 2005). In spring, univariate tests suggested that four of the five significant individual invertebrate species had a higher prevalence in coniferous than in mixed‐deciduous woodland diets (this rose to 6/7 in the analysis excluding rare species; see Table S5b,c). One of these, the green spruce aphid *Elatobium abietinum*, is an important pest of commercial plantations, suggesting great tits may help control their impacts as insect predators do (Day et al. 2006). Another species, the red‐line quaker moth *Agrochola lota* , reproduces on sallows and willows (*Salix* spp.), which are associated with the damp conditions on which conifer plantations are often established. The third, the July highflyer moth *Hydriomena furcata* , prefers woodland margins and hedgerows, while the fourth species, the hoverfly *Meliscaeva auricollis*, is found in all habitat types. Together these results suggest that while great tits undoubtedly feed on invertebrates adapted to conifer plantations, they also rely on species adapted to deciduous plants. These deciduous‐adapted invertebrates likely occur in hedges along site boundaries or on deciduous trees within the conifer plantations, for example in damp Salix scrub. In winter, community composition in diet also differed, including significant univariate tests for a greater prevalence of beech and gall wasps among faecal samples from mixed‐deciduous sites. Univariate tests also showed a greater prevalence of peanuts among those from conifer sites, the latter presumably reflecting greater feeder use because beech was less available in conifer sites (Table S5d,e).

Species richness also varied in the diets of birds from different habitats. When looking at the invertebrate diet alone, richness was similar between habitats in spring, which aligns with Shutt et al. (2020), who found that dietary richness in blue tits did not change with local tree community composition in spring. In winter, invertebrate richness was higher in mixed‐deciduous woodlands (see Table 2a; Figure 2b) but richness was similar between the habitat types when plant species were also included (Table 2c). Though previous studies suggest that food abundance may be lower in conifer‐dominated sites than deciduous (van Balen 1973; Sisask et al. 2010), our analyses suggest that diet diversity and perhaps overall nutrition are similar in the two habitat types. This is notwithstanding the fact that species vary in their nutritional quality and birds tend to select the species that are most nutritional in terms of macro‐ and micro‐nutrients (Razeng and Watson 2015).

Finally, we assume that the dietary items detected in the faecal samples reflect food collected within the same sites that the birds were captured. This is plausible because spring home range sizes in great tits are likely substantially smaller (e.g., radio tracking suggested a mean of 2.4 ha, Naguib et al. 2022) than the area of our study sites, which ranged from 10–25 ha. Additionally, in winter, tits show strong fidelity to feeding sites (Krištín and Kaňuch 2017). Nevertheless, birds sampled in either habitat type could have been feeding outside of the study sites, and this deserves further investigation.

### 4.4 Sex and Age

We report differences in dietary richness and community composition between sexes and ages. These differences often depended on an interaction between sex and age, and sometimes interactions with habitat or season. To our knowledge, this is one of the only studies to examine variation in species‐level components of the diet between sexes and ages in a fully grown passerine species (for examination of sex differences using OTUs see Jedlicka et al. 2017; da Silva et al. 2020 and for microscope analysis at the order level see Durst et al. 2008), and the only study to do so in this model species in avian ecology. Intraspecific variation can have important implications for the ecology and dynamics of populations. The extensive variation between sexes and age classes we report here, combined with the complexity of the diet, may partly explain why our ability to predict trends and population changes in response to food is typically limited (Newton 1998).

Most differences between sexes were in terms of dietary community composition. The bipartite network shows large overlap in species between the diets of males and females but also that many rare species are unique to each sex. In general, compositional differences in the diet in spring and in winter between ages were strongest as interactions with sex. Age differences in richness and composition were also present as interactions with season. First‐year birds had a higher invertebrate richness than adults but only in winter, which could be explained by first‐years being less experienced and therefore less selective foragers than adults (Goss‐Custard and Durell 1987; Daunt et al. 2007; Thornton 2008; Fayet et al. 2015). Juvenile birds undertake much trial and error when learning to select different prey types, especially in their first winter when invertebrate and plant food types are unknown and invertebrates may be difficult to find (Goss‐Custard and Durell 1987; Marchetti and Price 1989). This interpretation is also supported by the bipartite network which shows that first‐years consumed more unique taxa, and thus were accessing a broader range of resources than adults. Additionally, first‐year birds were far more likely to consume readily available beech mast in the winter than adults (96% vs. 52%) supporting the idea that first‐years are less experienced foragers than adults, who were presumably better able to forage on other natural but less easily located sources.

There was limited evidence for individual diet species driving compositional dietary differences, for either sex or age. This result could be linked to limited sample size, especially given the large correction terms involved with repeated testing for large numbers of prey species in the multivariate generalised linear model framework. However, an alternative is that most differences among cohorts reflect subtle differences in foraging mode, exposing the sexes or ages to different communities of predominantly rare invertebrates. This is suggested by the large number of unique prey species to each sex and each age (Figure 5). We suggest that foraging modes and microhabitats used, perhaps driven by differences in nutritional requirements (Reynolds and Perrins 2010), competitive ability (Svanbäck and Persson 2004; Svanbäck and Bolnick 2005, 2007) or beak morphology (Gosler 1987), could well provide an explanation for these dietary differences between the sexes and ages.

## 5 Conclusion

This study provides evidence of variation in the dietary richness and composition of great tits with respect to most of the variables considered. Specifically, it demonstrates that the foraging ecology of a generalist passerine species varies between ages and sexes, among habitat types, across seasons, and between years. We draw two general conclusions from these findings. First, the use of DNA metabarcoding opens up new avenues of research in taxa that have traditionally been difficult to study due to their size. Second, this dietary variation is likely to have important consequences for our understanding of how populations respond to environmental change. In tit species, for example, much focus has been put on how climate change could lead to a mismatch in the timing of breeding and the emergence of their main prey species, the winter moth, when provisioning offspring in oak woodlands. However, diet diversity in young, not to mention the impact of diet throughout the year among all age classes, has scarcely been considered. Failure to account for the diet variation we have identified here in research and management could inaccurately represent population‐level processes in these widespread generalist consumers.

## Author Contributions

**J. R. Coomes:** conceptualization (equal), data curation (lead), formal analysis (lead), methodology (equal), visualization (equal), writing – original draft (lead), writing – review and editing (lead). **J. P. Cuff:** formal analysis (supporting), visualization (supporting), writing – review and editing (equal). **M. S. Reichert:** supervision (supporting), visualization (supporting), writing – review and editing (equal). **G. L. Davidson:** data curation (supporting), supervision (supporting), visualization (supporting), writing – review and editing (equal). **W. O. C. Symondson:** methodology (supporting), supervision (supporting). **J. L. Quinn:** conceptualization (equal), funding acquisition (lead), methodology (equal), project administration (lead), supervision (lead), visualization (supporting), writing – original draft (supporting), writing – review and editing (equal).

## Conflicts of Interest

The authors declare no conflicts of interest.

## Supporting information


**Appendix S1.** Supporting Information.

## Data Availability

Data available from the Dryad Digital Repository: DOI: https://doi.org/10.5061/dryad.7h44j104p (Coomes et al., 2025).
