# Supplementary material for: Spatio‐Temporal Variation in Diet Among Age and Sex Cohorts of a Model Generalist Bird Species, the Great Tit Parus major : New Insights Revealed by DNA Metabarcoding
Source: Ecol Evol. 2025 Jul 14;15(7):e71565. doi: 10.1002/ece3.71565 (PMC12256774; doi:10.1002/ece3.71565)
Supplement: Supplementary file 1 — Appendix S1. Supporting Information. [file ECE3-15-e71565-s001.docx]

# Appendix

Table S1. List of the 12 study sites in the Bandon Valley area, Co. Cork (Ireland), where birds were captured and samples were taken. The main habitat type of each field site (mixed-deciduous forest vs. coniferous plantation), the area (hectares), the coordinates in decimal degrees (DD) and the number of faecal samples from each site that were collected are shown (includes duplicate samples).

| *The 12 study sites in the Bandon Valley* | | | | |
| --- | --- | --- | --- | --- |
| **Field site** | **Habitat type** | **Area (ha)** | **Coordinates (DD)** | **Number of samples** |
| Ballinphelic | Coniferous | 18 | 51.840074, -8.628249 | 25 |
| Castlebernard | Mixed | 17 | 51.741821, -8.773443 | 41 |
| Carrigeen | Coniferous | 15 | 51.821298, -8.814267 | 6 |
| Dunderrow | Mixed | 14 | 51.719711, -8.604451 | 47 |
| Dukes Wood | Mixed | 16 | 51.787396, -8.755946 | 44 |
| Farran | Coniferous | 11 | 51.700448, -8.805728 | 2 |
| Garretstown | Coniferous | 25 | 51.654770, -8.616293 | 13 |
| Inishannon | Mixed | 10 | 51.763048, -8.662415 | 22 |
| Kilbrittain | Mixed | 25 | 51.671880, -8.683837 | 41 |
| Lissarda | Coniferous | 15 | 51.858086, -8.859128 | 10 |
| Piercetown | Coniferous | 15 | 51.788888, -8.456197 | 5 |
| Shipool | Mixed | 18 | 51.737620, -8.631437 | 10 |

### DNA extraction

Extractions were carried out under a laminar flow hood that was sterilised with bleach and ethanol before and after each use. Tubes and pestles were autoclaved before use and all equipment was cleaned with bleach and placed under UV light for 20 min prior to extraction.

Up to 220 mg of each faecal sample was manually mixed with 1 ml InhibitEX Buffer (Qiagen, Manchester, UK) using a plastic pestle (500 µl at a time), vortexed for 3 min to homogenize the sample and incubated in a water bath for 15 min at 70°C. After another vortex for 2 min, samples were centrifuged for 3 min (every centrifuge step was at 13,000 rpm) to pellet the faecal particles. 400 µl of the faecal supernatant was added to 20 µl of proteinase K (Qiagen) and this was followed by adding 400 µl of Buffer AL (Qiagen). The samples were then vortexed for 15 s to form a homogenous solution. The samples were incubated at 70°C for 15 min and then centrifuged for 1 min to remove drops from the tube lid. 400 µl of ethanol (96 – 100%) was added to the lysate and the sample was vortexed for 30 s and centrifuged for 1 min. 600 µl of each lysate was added to a QIAmp spin column (NBS Biologicals) and was centrifuged for 1 min. The filtrate was then discarded. The remainder of the lysate was added to the spin column and centrifuged again for 1 min. Following this, 500 µl of the wash Buffer AW1 first, and Buffer AW2 second, was added to the spin column, with a 1 min centrifuge after each addition and the filtrate discarded. A further 3 min centrifuge followed this to eliminate Buffer AW2 carryover which could have caused problems in downstream analysis. 80 µl of Buffer AE was added to the spin column membrane, incubated for 5 min at room temperature and then centrifuged for 1 min to elute DNA. The solution was then passed through the spin column a second time to further concentrate the DNA and increase yield.


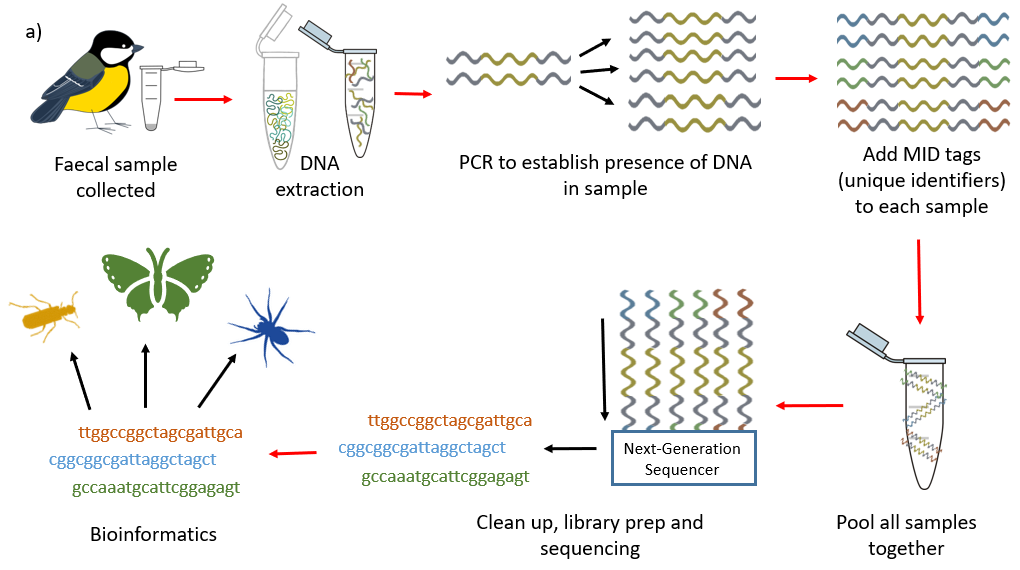

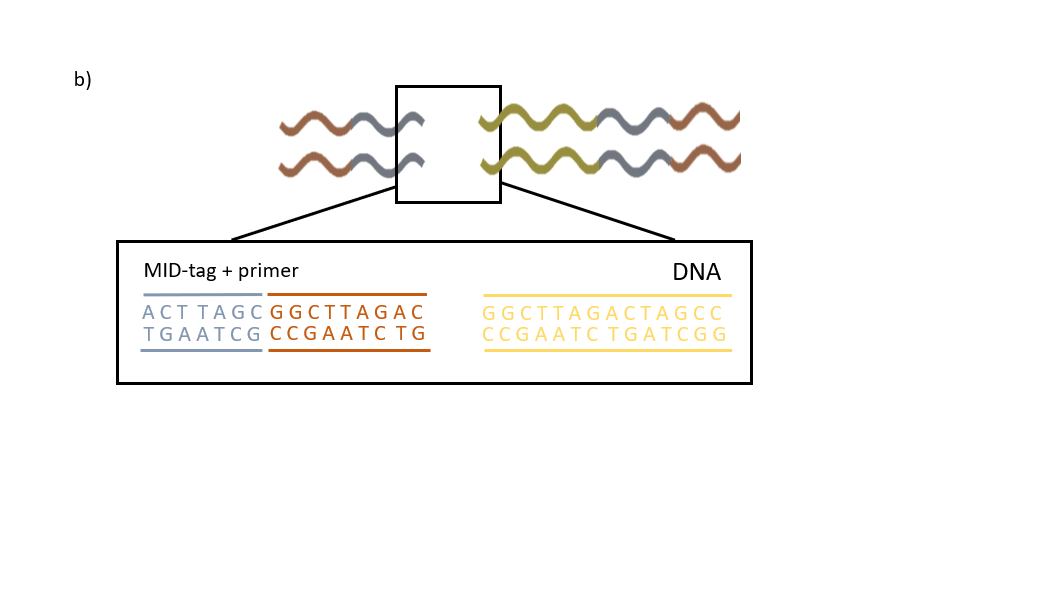


Figure S1. Illustration of the methods for determining diet are shown in panel a), and the addition of a unique identifier (MID-tag) is shown in panel b). Faecal samples were collected from the great tits and DNA was extracted from each sample. PCR was used to establish the presence of DNA. A unique identifier (MID-tag) was added to each sample using blunt-end ligation (shown in b) and the samples were pooled. Pooled samples were cleaned and sequenced using NGS (next-generation sequencing) to retrieve the sequences of each DNA fragment. The sequences were matched to a database (BOLD and GenBank) in a bioinformatics pipeline to determine the species present.

Table S2. Full species list for all the invertebrate species that were identified in the diet of the great tits. Percentage is the percentage frequency of occurrence. Data consists of 188 samples (122 in spring and 66 in winter) and does include duplicate samples where available (27 individuals had multiple samples). Green and blue highlights indicate significant univariate effects in spring and winter respectively (see Table S5a). Note that *Ochropleura implecta* is a North American species and is most likely to be the European *Ochropleura plecta* (flame shoulder moth) instead, though we do not know for certain.

| **Species** | **Family** | **Order** | **Group** | **No. samples overall** | **% overall** | **Spring** | **% Spring** | **Winter** | **% Winter** |
| --- | --- | --- | --- | --- | --- | --- | --- | --- | --- |
| *Operophtera.brumata* | Geometridae | Lepidoptera | moth | 84 | 45% | 84 | 69% | 0 | 0% |
| *Orthosia.cerasi* | Noctuidae | Lepidoptera | moth | 65 | 35% | 63 | 52% | 2 | 3% |
| *Amphipyra.pyramidea* | Noctuidae | Lepidoptera | moth | 41 | 22% | 39 | 32% | 2 | 3% |
| *Hydriomena.furcata* | Geometridae | Lepidoptera | moth | 40 | 21% | 39 | 32% | 1 | 2% |
| *Pandemis.cerasana* | Tortricidae | Lepidoptera | moth | 36 | 19% | 36 | 30% | 0 | 0% |
| *Anorthoa.munda* | Noctuidae | Lepidoptera | moth | 35 | 19% | 35 | 29% | 0 | 0% |
| *Elatobium.abietinum* | Aphididae | Hemiptera | aphid | 33 | 18% | 32 | 26% | 1 | 2% |
| *Syrphus.ribesii* | Syrphidae | Diptera | hoverfly | 32 | 17% | 31 | 25% | 1 | 2% |
| *Lozotaenia.forsterana* | Tortricidae | Lepidoptera | moth | 29 | 15% | 25 | 20% | 4 | 6% |
| *Melangyna.lasiophthalma* | Syrphidae | Diptera | hoverfly | 26 | 14% | 25 | 20% | 1 | 2% |
| *Anyphaena.accentuata* | Anyphaenidae | Araneae | spider | 24 | 13% | 14 | 11% | 10 | 15% |
| *Blastobasis.adustella* | Blastobasidae | Lepidoptera | moth | 25 | 13% | 16 | 13% | 9 | 14% |
| *Agriopis.marginaria* | Geometridae | Lepidoptera | moth | 23 | 12% | 23 | 19% | 0 | 0% |
| *Agrochola.lota* | Noctuidae | Lepidoptera | moth | 22 | 12% | 22 | 18% | 0 | 0% |
| *Eilema.depressum* | Erebidae | Lepidoptera | moth | 22 | 12% | 22 | 18% | 0 | 0% |
| *Acanthosoma.haemorrhoidale* | Acanthosomatidae | Hemiptera | shield bug | 21 | 11% | 6 | 5% | 15 | 23% |
| *Psychoda.phalaenoides* | Psychodidae | Diptera | moth fly | 21 | 11% | 11 | 9% | 10 | 15% |
| *Clubiona.reclusa* | Clubionidae | Araneae | sac spider | 21 | 11% | 21 | 17% | 0 | 0% |
| *Meliscaeva.auricollis* | Syrphidae | Diptera | hoverfly | 21 | 11% | 21 | 17% | 0 | 0% |
| *Epinotia.nisella* | Tortricidae | Lepidoptera | moth | 20 | 11% | 20 | 16% | 0 | 0% |
| *Neriene.montana* | Linyphiidae | Araneae | spider | 18 | 10% | 13 | 11% | 5 | 8% |
| *Tipula.oleracea* | Tipulidae | Diptera | crane fly | 18 | 10% | 15 | 12% | 3 | 5% |
| *Orthosia.cruda* | Noctuidae | Lepidoptera | moth | 18 | 10% | 18 | 15% | 0 | 0% |
| *Scathophaga.stercoraria* | Scathophagidae | Diptera | fly | 18 | 10% | 18 | 15% | 0 | 0% |
| *Neuroterus.quercusbaccarum* | Cynipidae | Hymenoptera | gall wasp | 17 | 9% | 0 | 0% | 17 | 26% |
| *Rhynchaenus.fagi* | Curculionidae | Coleoptera | weevil | 16 | 9% | 12 | 10% | 4 | 6% |
| *Syrphus.torvus* | Syrphidae | Diptera | hoverfly | 16 | 9% | 14 | 11% | 2 | 3% |
| *Araneus.triguttatus* | Araneidae | Araneae | spider | 16 | 9% | 15 | 12% | 1 | 2% |
| *Notocelia.uddmanniana* | Tortricidae | Lepidoptera | moth | 16 | 9% | 16 | 13% | 0 | 0% |
| *Thera.britannica* | Geometridae | Lepidoptera | moth | 16 | 9% | 16 | 13% | 0 | 0% |
| *Culicoides.impunctatus* | Ceratopogonidae | Diptera | midge | 15 | 8% | 15 | 12% | 0 | 0% |
| *Ptycholoma.lecheana* | Tortricidae | Lepidoptera | moth | 15 | 8% | 15 | 12% | 0 | 0% |
| *Amphorophora.rubi* | Aphididae | Hemiptera | aphid | 14 | 7% | 12 | 10% | 2 | 3% |
| *Ditula.angustiorana* | Tortricidae | Lepidoptera | moth | 14 | 7% | 13 | 11% | 1 | 2% |
| *Archips.podana* | Tortricidae | Lepidoptera | moth | 13 | 7% | 13 | 11% | 0 | 0% |
| *Dryobotodes.eremita* | Noctuidae | Lepidoptera | moth | 13 | 7% | 13 | 11% | 0 | 0% |
| *Phobocampe.bicingulata* | Ichneumonidae | Hymenoptera | parasitoid wasp | 13 | 7% | 13 | 11% | 0 | 0% |
| *Clubiona.comta* | Clubionidae | Araneae | sac spider | 12 | 6% | 2 | 2% | 10 | 15% |
| *Capua.vulgana* | Tortricidae | Lepidoptera | moth | 12 | 6% | 6 | 5% | 6 | 9% |
| *Amaurobius.similis* | Amaurobiidae | Araneae | spider | 11 | 6% | 5 | 4% | 6 | 9% |
| *Metellina.mengei* | Tetragnathidae | Araneae | spider | 12 | 6% | 9 | 7% | 3 | 5% |
| *Cheilosia.semifasciata* | Syrphidae | Diptera | hoverfly | 11 | 6% | 11 | 9% | 0 | 0% |
| *Cosmia.trapezina* | Noctuidae | Lepidoptera | moth | 11 | 6% | 11 | 9% | 0 | 0% |
| *Enytus.montanus* | Ichneumonidae | Hymenoptera | parasitoid wasp | 11 | 6% | 11 | 9% | 0 | 0% |
| *Glyptapanteles.porthetriae* | Braconidae | Hymenoptera | parasitoid wasp | 11 | 6% | 11 | 9% | 0 | 0% |
| *Mesapamea.secalella* | Noctuidae | Lepidoptera | moth | 11 | 6% | 11 | 9% | 0 | 0% |
| *Sitobion.sp.* | Aphididae | Hemiptera | aphid | 11 | 6% | 11 | 9% | 0 | 0% |
| *Amaurobius.fenestralis* | Amaurobiidae | Araneae | spider | 10 | 5% | 0 | 0% | 10 | 15% |
| *Clubiona.terrestris* | Clubionidae | Araneae | sac spider | 9 | 5% | 0 | 0% | 9 | 14% |
| *Bradysia.placida* | Sciaridae | Diptera | gnat | 9 | 5% | 1 | 1% | 8 | 12% |
| *Lithophane.socia* | Noctuidae | Lepidoptera | moth | 9 | 5% | 3 | 2% | 6 | 9% |
| *Phlogophora.meticulosa* | Noctuidae | Lepidoptera | moth | 10 | 5% | 5 | 4% | 5 | 8% |
| *Agonopterix.nervosa* | Depressariidae | Lepidoptera | moth | 10 | 5% | 10 | 8% | 0 | 0% |
| *Aleiodes.pictus* | Braconidae | Hymenoptera | parasitoid wasp | 10 | 5% | 10 | 8% | 0 | 0% |
| *Petrophora.chlorosata* | Geometridae | Lepidoptera | moth | 10 | 5% | 10 | 8% | 0 | 0% |
| *Cotesia.spuria* | Braconidae | Hymenoptera | parasitoid wasp | 9 | 5% | 9 | 7% | 0 | 0% |
| *Lonchoptera.lutea* | Lonchopteridae | Diptera | fly | 8 | 4% | 0 | 0% | 8 | 12% |
| *Ochropleura.implecta* | Noctuidae | Lepidoptera | moth | 8 | 4% | 0 | 0% | 8 | 12% |
| *Herminia.grisealis* | Erebidae | Lepidoptera | moth | 8 | 4% | 1 | 1% | 7 | 11% |
| *Phaonia.tuguriorum* | Muscidae | Diptera | fly | 7 | 4% | 0 | 0% | 7 | 11% |
| *Polia.nebulosa* | Noctuidae | Lepidoptera | moth | 7 | 4% | 0 | 0% | 7 | 11% |
| *Nalassus.sp.* | Tenebrionidae | Coleoptera | beetle | 7 | 4% | 2 | 2% | 5 | 8% |
| *Cyclophora.linearia* | Geometridae | Lepidoptera | moth | 8 | 4% | 4 | 3% | 4 | 6% |
| *Meliscaeva.cinctella* | Syrphidae | Diptera | hoverfly | 8 | 4% | 4 | 3% | 4 | 6% |
| *Rhynchaenus.quercus* | Curculionidae | Coleoptera | weevil | 8 | 4% | 4 | 3% | 4 | 6% |
| *Deileptenia.ribeata* | Geometridae | Lepidoptera | moth | 8 | 4% | 6 | 5% | 2 | 3% |
| *Orthosia.gothica* | Noctuidae | Lepidoptera | moth | 8 | 4% | 7 | 6% | 1 | 2% |
| *Orthosia.incerta* | Noctuidae | Lepidoptera | moth | 8 | 4% | 7 | 6% | 1 | 2% |
| *Spilarctia.luteum* | Erebidae | Lepidoptera | moth | 8 | 4% | 7 | 6% | 1 | 2% |
| *Macrosiphum.funestum* | Aphididae | Hemiptera | aphid | 7 | 4% | 6 | 5% | 1 | 2% |
| *Colotois.pennaria* | Geometridae | Lepidoptera | moth | 8 | 4% | 8 | 7% | 0 | 0% |
| *Cyclosa.conica* | Araneidae | Araneae | spider | 8 | 4% | 8 | 7% | 0 | 0% |
| *Ectropis.crepuscularia* | Geometridae | Lepidoptera | moth | 8 | 4% | 8 | 7% | 0 | 0% |
| *Helophilus.pendulus* | Syrphidae | Diptera | hoverfly | 8 | 4% | 8 | 7% | 0 | 0% |
| *Pandemis.heparana* | Tortricidae | Lepidoptera | moth | 8 | 4% | 8 | 7% | 0 | 0% |
| *Scoliopteryx.libatrix* | Erebidae | Lepidoptera | moth | 8 | 4% | 8 | 7% | 0 | 0% |
| *Acrobasis.advenella* | Pyralidae | Lepidoptera | moth | 7 | 4% | 7 | 6% | 0 | 0% |
| *Conistra.vaccinii* | Noctuidae | Lepidoptera | moth | 7 | 4% | 7 | 6% | 0 | 0% |
| *Hypena.proboscidalis* | Erebidae | Lepidoptera | moth | 7 | 4% | 7 | 6% | 0 | 0% |
| *Plectiscus.impurator* | Ichneumonidae | Hymenoptera | parasitoid wasp | 6 | 3% | 0 | 0% | 6 | 9% |
| *Mesopolobus.tibialis* | Pteromalidae | Hymenoptera | fly | 5 | 3% | 0 | 0% | 5 | 8% |
| *Clubiona.brevipes* | Clubionidae | Araneae | sac spider | 6 | 3% | 2 | 2% | 4 | 6% |
| *Lepthyphantes.minutus* | Linyphiidae | Araneae | spider | 5 | 3% | 1 | 1% | 4 | 6% |
| *Syndemis.musculana* | Tortricidae | Lepidoptera | moth | 5 | 3% | 1 | 1% | 4 | 6% |
| *Chrysotropia.ciliata* | Chrysopidae | Neuroptera | lacewing | 5 | 3% | 2 | 2% | 3 | 5% |
| *Baetis.rhodani* | Baetidae | Ephemeroptera | mayfly | 6 | 3% | 4 | 3% | 2 | 3% |
| *Aphidius.urticae* | Braconidae | Hymenoptera | parasitoid wasp | 5 | 3% | 3 | 2% | 2 | 3% |
| *Zeiraphera.ratzeburgiana* | Tortricidae | Lepidoptera | moth | 5 | 3% | 3 | 2% | 2 | 3% |
| *Syrphus.opinator* | Syrphidae | Diptera | hoverfly | 6 | 3% | 5 | 4% | 1 | 2% |
| *Neriene.peltata* | Linyphiidae | Araneae | spider | 5 | 3% | 4 | 3% | 1 | 2% |
| *Bombus.lucorum* | Apidae | Hymenoptera | bee | 6 | 3% | 6 | 5% | 0 | 0% |
| *Psychoda.trinodulosa* | Psychodidae | Diptera | moth fly | 6 | 3% | 6 | 5% | 0 | 0% |
| *Quercusia.quercus* | Lycaenidae | Lepidoptera | butterfly | 6 | 3% | 6 | 5% | 0 | 0% |
| *Agonopterix.heracliana* | Depressariidae | Lepidoptera | moth | 5 | 3% | 5 | 4% | 0 | 0% |
| *Cinara.pilicornis* | Aphididae | Hemiptera | aphid | 5 | 3% | 5 | 4% | 0 | 0% |
| *Gymnoscelis.rufifasciata* | Geometridae | Lepidoptera | moth | 5 | 3% | 5 | 4% | 0 | 0% |
| *Hydriomena.ruberata* | Geometridae | Lepidoptera | moth | 5 | 3% | 5 | 4% | 0 | 0% |
| *Lehmannia.marginata* | Limacidae | Pulmonata | slug | 5 | 3% | 5 | 4% | 0 | 0% |
| *Protapanteles.fulvipes* | Braconidae | Hymenoptera | parasitoid wasp | 5 | 3% | 5 | 4% | 0 | 0% |
| *Pygostolus.falcatus* | Braconidae | Hymenoptera | parasitoid wasp | 5 | 3% | 5 | 4% | 0 | 0% |
| *Rhagium.bifasciatum* | Cerambycidae | Coleoptera | beetle | 5 | 3% | 5 | 4% | 0 | 0% |
| *Diurnea.fagella* | Chimabachidae | Lepidoptera | moth | 4 | 2% | 0 | 0% | 4 | 6% |
| *Phyllonorycter.hilarella* | Gracillariidae | Lepidoptera | moth | 4 | 2% | 0 | 0% | 4 | 6% |
| *Ophion.mocsaryi* | Ichneumonidae | Hymenoptera | parasitoid wasp | 4 | 2% | 1 | 1% | 3 | 5% |
| *Biston.betularia* | Geometridae | Lepidoptera | moth | 4 | 2% | 2 | 2% | 2 | 3% |
| *Peristenus.sp.* | Braconidae | Hymenoptera | parasitoid wasp | 4 | 2% | 2 | 2% | 2 | 3% |
| *Calliteara.pudibunda* | Erebidae | Lepidoptera | moth | 3 | 2% | 1 | 1% | 2 | 3% |
| *Pardosa.amentata* | Lycosidae | Araneae | spider | 3 | 2% | 1 | 1% | 2 | 3% |
| *Hemerobius.micans* | Hemerobiidae | Neuroptera | lacewing | 3 | 2% | 2 | 2% | 1 | 2% |
| *Ephedrus.lacertosus* | Braconidae | Hymenoptera | parasitoid wasp | 4 | 2% | 4 | 3% | 0 | 0% |
| *Euura.viduata* | Tenthredinidae | Hymenoptera | sawfly | 4 | 2% | 4 | 3% | 0 | 0% |
| *Melolontha.melolontha* | Scarabaeidae | Coleoptera | beetle | 4 | 2% | 4 | 3% | 0 | 0% |
| *Polydrusus.undatus* | Curculionidae | Coleoptera | weevil | 4 | 2% | 4 | 3% | 0 | 0% |
| *Sussaba.aciculata* | Ichneumonidae | Hymenoptera | parasitoid wasp | 4 | 2% | 4 | 3% | 0 | 0% |
| *Syrphoctonus.tarsatorius* | Ichneumonidae | Hymenoptera | parasitoid wasp | 4 | 2% | 4 | 3% | 0 | 0% |
| *Aglais.urticae* | Nymphalidae | Lepidoptera | butterfly | 3 | 2% | 3 | 2% | 0 | 0% |
| *Cinara.pruinosa* | Aphididae | Hemiptera | aphid | 3 | 2% | 3 | 2% | 0 | 0% |
| *Coleophora.serratella* | Coleophoridae | Lepidoptera | moth | 3 | 2% | 3 | 2% | 0 | 0% |
| *Eriocrania.cicatricella* | Eriocraniidae | Lepidoptera | moth | 3 | 2% | 3 | 2% | 0 | 0% |
| *Euproctis.similis* | Erebidae | Lepidoptera | moth | 3 | 2% | 3 | 2% | 0 | 0% |
| *Ochropacha.duplaris* | Drepanidae | Lepidoptera | moth | 3 | 2% | 3 | 2% | 0 | 0% |
| *Pammene.giganteana* | Tortricidae | Lepidoptera | moth | 3 | 2% | 3 | 2% | 0 | 0% |
| *Pterophorus.pentadactyla* | Pterophoridae | Lepidoptera | moth | 3 | 2% | 3 | 2% | 0 | 0% |
| *Lacanobia.oleracea* | Noctuidae | Lepidoptera | moth | 2 | 1% | 0 | 0% | 2 | 3% |
| *Noctua.pronuba* | Noctuidae | Lepidoptera | moth | 2 | 1% | 0 | 0% | 2 | 3% |
| *Spilosoma.lubricipedum* | Erebidae | Lepidoptera | moth | 2 | 1% | 0 | 0% | 2 | 3% |
| *Trochosa.ruricola* | Lycosidae | Araneae | spider | 2 | 1% | 0 | 0% | 2 | 3% |
| *Xestia.triangulum* | Noctuidae | Lepidoptera | moth | 2 | 1% | 0 | 0% | 2 | 3% |
| *Hepialus.fusconebulosus* | Hepialidae | Lepidoptera | moth | 2 | 1% | 1 | 1% | 1 | 2% |
| *Syrrhizus.sp.* | Braconidae | Hymenoptera | parasitoid wasp | 2 | 1% | 1 | 1% | 1 | 2% |
| *Chalarus.fimbriatus* | Pipunculidae | Diptera | fly | 1 | 1% | 0 | 0% | 1 | 2% |
| *Coelichneumon.deliratorius* | Ichneumonidae | Hymenoptera | parasitoid wasp | 1 | 1% | 0 | 0% | 1 | 2% |
| *Crepidodera.fulvicornis* | Chrysomelidae | Coleoptera | beetle | 1 | 1% | 0 | 0% | 1 | 2% |
| *Drapetisca.socialis* | Linyphiidae | Araneae | spider | 1 | 1% | 0 | 0% | 1 | 2% |
| *Elachiptera.decipiens* | Chloropidae | Diptera | fly | 1 | 1% | 0 | 0% | 1 | 2% |
| *Epiphyas.postvittana* | Tortricidae | Lepidoptera | moth | 1 | 1% | 0 | 0% | 1 | 2% |
| *Forficula.sp.* | Forficulidae | Dermaptera | earwig | 1 | 1% | 0 | 0% | 1 | 2% |
| *Meteorus.consimilis* | Braconidae | Hymenoptera | parasitoid wasp | 1 | 1% | 0 | 0% | 1 | 2% |
| *Neon.reticulatus* | Salticidae | Araneae | spider | 1 | 1% | 0 | 0% | 1 | 2% |
| *Phragmatobia.fuliginosa* | Erebidae | Lepidoptera | moth | 1 | 1% | 0 | 0% | 1 | 2% |
| *Rhopalosiphum.padi* | Aphididae | Hemiptera | aphid | 1 | 1% | 0 | 0% | 1 | 2% |
| *Abrostola.tripartita* | Noctuidae | Lepidoptera | moth | 2 | 1% | 2 | 2% | 0 | 0% |
| *Acleris.laterana* | Tortricidae | Lepidoptera | moth | 2 | 1% | 2 | 2% | 0 | 0% |
| *Aphidius.rosae* | Braconidae | Hymenoptera | parasitoid wasp | 2 | 1% | 2 | 2% | 0 | 0% |
| *Colostygia.pectinataria* | Geometridae | Lepidoptera | moth | 2 | 1% | 2 | 2% | 0 | 0% |
| *Earinus.sp.* | Braconidae | Hymenoptera | parasitoid wasp | 2 | 1% | 2 | 2% | 0 | 0% |
| *Syrphophilus.tricinctorius* | Ichneumonidae | Hymenoptera | parasitoid wasp | 2 | 1% | 2 | 2% | 0 | 0% |
| *Tetragnatha.obtusa* | Tetragnathidae | Araneae | spider | 2 | 1% | 2 | 2% | 0 | 0% |
| *Zele.deceptor* | Braconidae | Hymenoptera | parasitoid wasp | 2 | 1% | 2 | 2% | 0 | 0% |
| *Athous.sp.* | Elateridae | Coleoptera | beetle | 1 | 1% | 1 | 1% | 0 | 0% |
| *Celypha.lacunana* | Tortricidae | Lepidoptera | moth | 1 | 1% | 1 | 1% | 0 | 0% |
| *Dictyna.arundinacea* | Dictynidae | Araneae | spider | 1 | 1% | 1 | 1% | 0 | 0% |
| *Eupithecia.vulgata* | Geometridae | Lepidoptera | moth | 1 | 1% | 1 | 1% | 0 | 0% |
| *Mesoleuca.albicillata* | Geometridae | Lepidoptera | moth | 1 | 1% | 1 | 1% | 0 | 0% |
| *Metopolophium.sp.* | Aphididae | Hemiptera | aphid | 1 | 1% | 1 | 1% | 0 | 0% |
| *Nomada.marshamella* | Apidae | Hymenoptera | bee | 1 | 1% | 1 | 1% | 0 | 0% |
| *Pararge.aegeria.aegeria* | Nymphalidae | Lepidoptera | butterfly | 1 | 1% | 1 | 1% | 0 | 0% |
| *Phratora.vulgatissima* | Chrysomelidae | Coleoptera | beetle | 1 | 1% | 1 | 1% | 0 | 0% |
| *Syrphoctonus.alaskensis* | Ichneumonidae | Hymenoptera | parasitoid wasp | 1 | 1% | 1 | 1% | 0 | 0% |
| *Xestia.xanthographa* | Noctuidae | Lepidoptera | moth | 1 | 1% | 0 | 0% | 1 | 2% |

| Table S3. Full species list of plants identified in the winter diet of the great tit. %FOO is the percentage frequency of occurrence (N = 106). | | | | | | | |
| --- | --- | --- | --- | --- | --- | --- | --- |
| **Species** | **Common name** | **No. samples** | **% FOO overall** | **Deciduous (n)** | **% FOO Deciduous** | **Conifer (n)** | **% FOO Conifer** |
| *Fagus.sp.* | Beech | 94 | 89% | 83 | 94% | 11 | 61% |
| *Helianthus.sp.* | Sunflower | 80 | 75% | 69 | 78% | 11 | 61% |
| *Rubus.sp.* | Fruit berry bushes | 48 | 45% | 36 | 41% | 12 | 67% |
| *Arachis.sp.* | Peanut | 41 | 39% | 28 | 32% | 13 | 72% |
| *Acer.pseudoplatanus* | Sycamore | 9 | 8% | 8 | 9% | 1 | 6% |
| *Plantago.sp.* | Plantain | 9 | 8% | 8 | 9% | 1 | 6% |
| *Quercus.petraea* | Irish/Sessile oak | 9 | 8% | 6 | 7% | 3 | 17% |
| *Ulmus.sp.* | Elm | 9 | 8% | 7 | 8% | 2 | 11% |
| *Viola.acuminata* | Acuminate violet | 8 | 8% | 7 | 8% | 1 | 6% |
| *Chamaenerion.sp.* | Wilowherb / fireweed | 7 | 7% | 6 | 7% | 1 | 6% |
| *Taxus.baccata* | Yew | 7 | 7% | 7 | 8% | 0 | 0% |
| *Hedera.helix* | Ivy | 6 | 6% | 5 | 6% | 1 | 6% |
| *Prunus.dulcis* | Almond | 6 | 6% | 5 | 6% | 1 | 6% |
| *Sonchus.oleraceus* | Common sowthistle | 6 | 6% | 5 | 6% | 1 | 6% |
| *Fraxinus.angustifolia* | Ash | 4 | 4% | 4 | 5% | 0 | 0% |
| *Ilex.sp.* | Holly | 4 | 4% | 4 | 5% | 0 | 0% |
| *Urtica.sp.* | Nettle | 4 | 4% | 3 | 3% | 1 | 6% |
| *Acer.platanoides* | Norway maple | 3 | 3% | 3 | 3% | 0 | 0% |
| *Alnus.sp.* | Alder | 3 | 3% | 3 | 3% | 0 | 0% |
| *Brassica.sp.* | Cabbage / Mustard | 3 | 3% | 1 | 1% | 2 | 11% |
| *Picea.sp.* | Spruce | 3 | 3% | 1 | 1% | 2 | 11% |
| *Prunus.domestica* | Plum | 3 | 3% | 2 | 2% | 1 | 6% |
| *Salix.sp.* | Willow | 3 | 3% | 1 | 1% | 2 | 11% |
| *Viola.reichenbachiana* | Early dog-violet | 3 | 3% | 2 | 2% | 1 | 6% |
| *Geranium.robertianum* | Herb-robert | 2 | 2% | 1 | 1% | 1 | 6% |
| *Jacobaea.vulgaris* | Ragwort | 2 | 2% | 1 | 1% | 1 | 6% |
| *Juglans.regia* | Walnut | 2 | 2% | 1 | 1% | 1 | 6% |
| *Pinus.sp.* | Pine | 2 | 2% | 1 | 1% | 1 | 6% |
| *Quercus.robur* | English/Pedunculate oak | 2 | 2% | 2 | 2% | 0 | 0% |
| *Agrostis.sp.* | Bentgrass | 1 | 1% | 1 | 1% | 0 | 0% |
| *Avena.fatua* | Wild oat | 1 | 1% | 1 | 1% | 0 | 0% |
| *Calluna.vulgaris* | Ling heather | 1 | 1% | 0 | 0% | 1 | 6% |
| *Dactylis.glomerata* | Cock’s-foot / cat grass | 1 | 1% | 1 | 1% | 0 | 0% |
| *Embothrium.coccineum* | Chilean Flame tree | 1 | 1% | 1 | 1% | 0 | 0% |
| *Galium.aparine* | Stickyweed | 1 | 1% | 0 | 0% | 1 | 6% |
| *Helosciadium.sp.* | Water cress | 1 | 1% | 1 | 1% | 0 | 0% |
| *Hordeum.vulgare* | Barley | 1 | 1% | 0 | 0% | 1 | 6% |
| *Ligustrum.vulgare* | Privet | 1 | 1% | 1 | 1% | 0 | 0% |
| *Linum.usitatissimum* | Linseed / flax | 1 | 1% | 1 | 1% | 0 | 0% |
| *Quercus.macranthera* | Persian oak | 1 | 1% | 1 | 1% | 0 | 0% |
| *Rhododendron.caucasicum* | Rhododendron | 1 | 1% | 1 | 1% | 0 | 0% |
| *Rosa.sp.* | Rose | 1 | 1% | 0 | 0% | 1 | 6% |
| *Sambucus.sp.* | Elderberry | 1 | 1% | 0 | 0% | 1 | 6% |
| *Scrophularia.balbisii* | Figwort | 1 | 1% | 1 | 1% | 0 | 0% |
| *Triticum.sp.* | Wheat | 1 | 1% | 1 | 1% | 0 | 0% |
| *Umbilicus.horizontalis* | Horizontal navelwort | 1 | 1% | 1 | 1% | 0 | 0% |
| *Veronica.persica* | Birdeye speedwell | 1 | 1% | 1 | 1% | 0 | 0% |


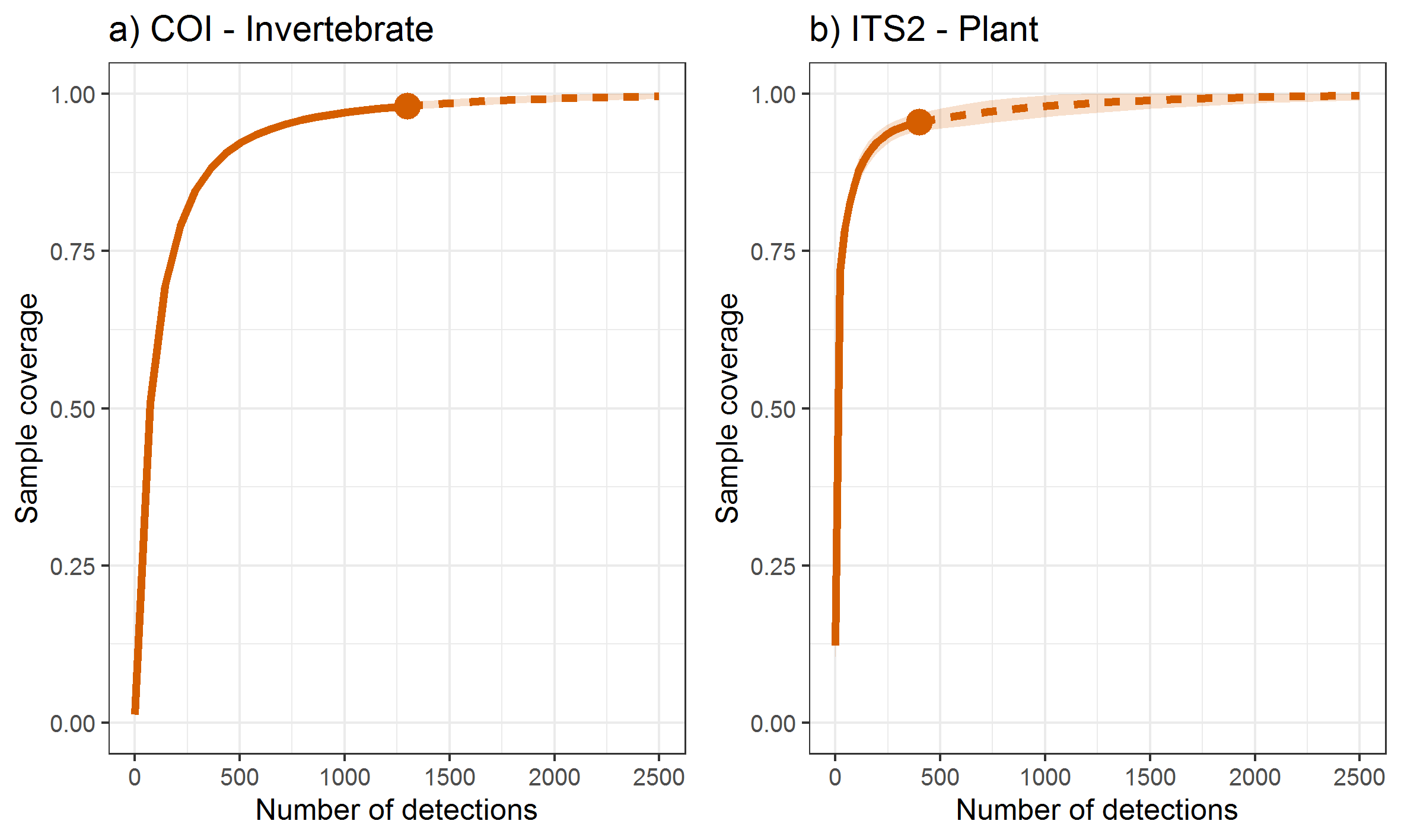
Figure S2. Sample coverage calculated for a) invertebrate metabarcoding (COI) and for b) plant metabarcoding (ITS2). Solid lines represent observed diversity, and dashed lines extrapolated diversity. Light zones surrounding lines denote 95% confidence intervals.


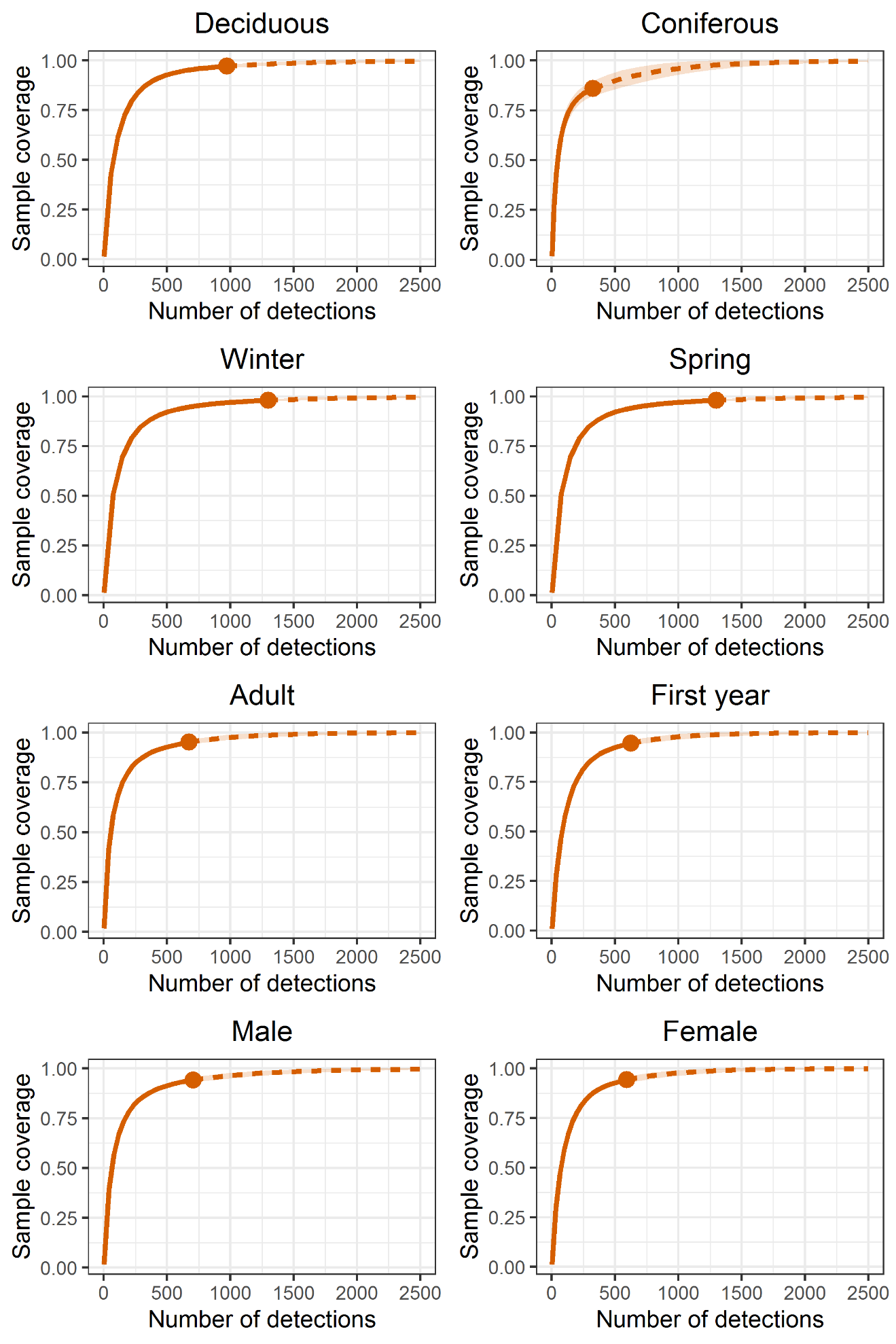
Figure S3. Sample coverage of the population dietary diversity detected via invertebrate metabarcoding in different habitats, seasons, ages and sexes. Solid lines represent observed diversity, and dashed lines extrapolated diversity. Light zones surrounding lines denote 95% confidence intervals. Completeness was high in all cases, but slightly lower for winter and coniferous diets.

Table S4. Full species list for all the invertebrate and plant species that were identified in the diet of the great tits in winter. Percentage frequency of occurrence is shown. Data consists of 109 samples and does not contain any duplicate samples from the same bird. The N of 109 here is larger than the 106 for the plants only as the diets of three birds contained invertebrate material only.

| **Species** | **Family** | **Order** | **Invert group / Plant species** | **% Adult** | **% First year** | **% Female** | **% Male** | **% All** |
| --- | --- | --- | --- | --- | --- | --- | --- | --- |
| *Fagus.sp.* | Fagaceae | Fagales | beech | 52% | 96% | 90% | 81% | 94% |
| *Helianthus.sp.* | Asteraceae | Asterales | sunflower | 56% | 79% | 75% | 71% | 81% |
| *Rubus.sp.* | Rosaceae | Rosales | fruit berry bush | 44% | 44% | 43% | 46% | 48% |
| *Arachis.sp.* | Fabaceae | Fabales | peanut | 48% | 35% | 34% | 42% | 42% |
| *Neuroterus.quercusbaccarum* | Cynipidae | Hymenoptera | gall wasp | 8% | 18% | 21% | 8% | 18% |
| *Acanthosoma.haemorrhoidale* | Acanthosomatidae | Hemiptera | shield bug | 12% | 14% | 11% | 17% | 15% |
| *Amaurobius.fenestralis* | Amaurobiidae | Araneae | spider | 0% | 12% | 11% | 6% | 11% |
| *Clubiona.comta* | Clubionidae | Araneae | sac spider | 4% | 11% | 11% | 6% | 11% |
| *Psychoda.phalaenoides* | Psychodidae | Diptera | moth fly | 8% | 10% | 7% | 13% | 11% |
| *Clubiona.terrestris* | Clubionidae | Araneae | sac spider | 0% | 11% | 8% | 8% | 10% |
| *Plantago.sp.* | Plantaginaceae | Lamiales | plantain | 0% | 11% | 7% | 10% | 10% |
| *Acer.pseudoplatanus* | Sapindaceae | Sapindales | sycamore | 4% | 10% | 8% | 8% | 10% |
| *Ulmus.sp.* | Ulmaceae | Rosales | elm | 4% | 10% | 10% | 6% | 10% |
| *Anyphaena.accentuata* | Anyphaenidae | Araneae | spider | 16% | 7% | 7% | 13% | 10% |
| *Quercus.petraea* | Fagaceae | Fagales | Irish/Sessile oak | 16% | 6% | 8% | 8% | 10% |
| *Bradysia.placida* | Sciaridae | Diptera | gnat | 0% | 10% | 10% | 4% | 9% |
| *Lonchoptera.lutea* | Lonchopteridae | Diptera | fly | 0% | 10% | 8% | 6% | 9% |
| *Ochropleura.implecta* | Noctuidae | Lepidoptera | moth | 0% | 10% | 8% | 6% | 9% |
| *Blastobasis.adustella* | Blastobasidae | Lepidoptera | moth | 12% | 7% | 11% | 4% | 9% |
| *Viola.acuminata* | Violaceae | Malpighiales | Acuminate violet | 8% | 7% | 7% | 8% | 8% |
| *Chamaenerion.sp.* | Onagaceae | Myrtales | Willowherb/fireweed | 8% | 6% | 7% | 6% | 8% |
| *Phaonia.tuguriorum* | Muscidae | Diptera | fly | 8% | 6% | 11% | 0% | 8% |
| *Herminia.grisealis* | Erebidae | Lepidoptera | moth | 0% | 8% | 8% | 4% | 7% |
| *Taxus.baccata* | Taxaceae | Cupressales | yew | 0% | 8% | 3% | 10% | 7% |
| *Polia.nebulosa* | Noctuidae | Lepidoptera | moth | 4% | 7% | 8% | 4% | 7% |
| *Lithophane.socia* | Noctuidae | Lepidoptera | moth | 4% | 6% | 8% | 2% | 7% |
| *Plectiscus.impurator* | Ichneumonidae | Hymenoptera | parasitoid wasp | 4% | 6% | 7% | 4% | 7% |
| *Prunus.dulcis* | Rosaceae | Rosales | almond | 4% | 6% | 10% | 0% | 7% |
| *Sonchus.oleraceus* | Asteraceae | Asterales | sowthistle | 4% | 6% | 8% | 2% | 7% |
| *Amaurobius.similis* | Amaurobiidae | Araneae | spider | 0% | 7% | 7% | 4% | 6% |
| *Capua.vulgana* | Tortricidae | Lepidoptera | moth | 0% | 7% | 7% | 4% | 6% |
| *Hedera.helix* | Araliaceae | Apiales | ivy | 0% | 7% | 8% | 2% | 6% |
| *Nalassus.sp.* | Tenebrionidae | Coleoptera | beetle | 0% | 6% | 7% | 2% | 6% |
| *Mesopolobus.tibialis* | Pteromalidae | Hymenoptera | fly | 4% | 5% | 7% | 2% | 6% |
| *Neriene.montana* | Linyphiidae | Araneae | spider | 4% | 5% | 7% | 2% | 6% |
| *Cyclophora.linearia* | Geometridae | Lepidoptera | moth | 0% | 5% | 5% | 2% | 5% |
| *Diurnea.fagella* | Chimabachidae | Lepidoptera | moth | 0% | 5% | 3% | 4% | 5% |
| *Fraxinus.angustifolia* | Oleaceae | Lamiales | ash | 0% | 5% | 7% | 0% | 5% |
| *Lepthyphantes.minutus* | Linyphiidae | Araneae | spider | 0% | 5% | 5% | 2% | 5% |
| *Meliscaeva.cinctella* | Syrphidae | Diptera | hoverfly | 0% | 5% | 5% | 2% | 5% |
| *Phyllonorycter.hilarella* | Gracillariidae | Lepidoptera | moth | 0% | 5% | 7% | 0% | 5% |
| *Rhynchaenus.fagi* | Curculionidae | Coleoptera | weevil | 0% | 5% | 5% | 2% | 5% |
| *Rhynchaenus.quercus* | Curculionidae | Coleoptera | weevil | 0% | 5% | 5% | 2% | 5% |
| *Syndemis.musculana* | Tortricidae | Lepidoptera | moth | 0% | 5% | 5% | 2% | 5% |
| *Urtica.sp.* | Urticaceae | Rosales | nettle | 0% | 5% | 5% | 2% | 5% |
| *Clubiona.brevipes* | Clubionidae | Araneae | sac spider | 4% | 4% | 2% | 6% | 5% |
| *Ilex.sp.* | Aquifoliaceae | Aquifoliales | holly | 4% | 4% | 3% | 4% | 5% |
| *Lozotaenia.forsterana* | Tortricidae | Lepidoptera | moth | 4% | 4% | 3% | 4% | 5% |
| *Phlogophora.meticulosa* | Noctuidae | Lepidoptera | moth | 16% | 1% | 7% | 2% | 5% |
| *Acer.platanoides* | Sapindaceae | Sapindales | Norway maple | 0% | 4% | 3% | 2% | 4% |
| *Metellina.mengei* | Tetragnathidae | Araneae | spider | 0% | 4% | 2% | 4% | 4% |
| *Ophion.mocsaryi* | Ichneumonidae | Hymenoptera | parasitoid wasp | 0% | 4% | 0% | 6% | 4% |
| *Alnus.sp.* | Betulaceae | Fagales | alder | 4% | 2% | 0% | 6% | 3% |
| *Chrysotropia.ciliata* | Chrysopidae | Neuroptera | lacewing | 4% | 2% | 5% | 0% | 3% |
| *Prunus.domestica* | Rosaceae | Rosales | plum | 4% | 2% | 2% | 4% | 3% |
| *Tipula.oleracea* | Tipulidae | Diptera | crane fly | 4% | 2% | 3% | 2% | 3% |
| *Viola.reichenbachiana* | Violaceae | Malpighiales | Early dog-violet | 4% | 2% | 2% | 4% | 3% |
| *Brassica.sp.* | Brassicaceae | Brassicales | Cabbage / mustard | 8% | 1% | 2% | 4% | 3% |
| *Picea.sp.* | Pinaceae | Pinales | spruce | 8% | 1% | 2% | 4% | 3% |
| *Salix.sp.* | Salicaceae | Malpighiales | willow | 8% | 1% | 3% | 2% | 3% |
| *Amphorophora.rubi* | Aphididae | Hemiptera | aphid | 0% | 2% | 2% | 2% | 2% |
| *Aphidius.urticae* | Braconidae | Hymenoptera | parasitoid wasp | 0% | 2% | 3% | 0% | 2% |
| *Baetis.rhodani* | Baetidae | Ephemeroptera | mayfly | 0% | 2% | 2% | 2% | 2% |
| *Biston.betularia* | Geometridae | Lepidoptera | moth | 0% | 2% | 3% | 0% | 2% |
| *Calliteara.pudibunda* | Erebidae | Lepidoptera | moth | 0% | 2% | 2% | 2% | 2% |
| *Geranium.robertianum* | Geraniaceae | Geraniales | Herb-robert | 0% | 2% | 0% | 4% | 2% |
| *Noctua.pronuba* | Noctuidae | Lepidoptera | moth | 0% | 2% | 2% | 2% | 2% |
| *Peristenus.sp.* | Braconidae | Hymenoptera | parasitoid wasp | 0% | 2% | 3% | 0% | 2% |
| *Syrphus.torvus* | Syrphidae | Diptera | hoverfly | 0% | 2% | 2% | 2% | 2% |
| *Trochosa.ruricola* | Lycosidae | Araneae | spider | 0% | 2% | 2% | 2% | 2% |
| *Zeiraphera.ratzeburgiana* | Tortricidae | Lepidoptera | moth | 0% | 2% | 2% | 2% | 2% |
| *Amphipyra.pyramidea* | Noctuidae | Lepidoptera | moth | 4% | 1% | 2% | 2% | 2% |
| *Deileptenia.ribeata* | Geometridae | Lepidoptera | moth | 4% | 1% | 3% | 0% | 2% |
| *Jacobaea.vulgaris* | Asteraceae | Asterales | ragwort | 4% | 1% | 3% | 0% | 2% |
| *Juglans.regia* | Juglandaceae | Fagales | Walnut | 4% | 1% | 2% | 2% | 2% |
| *Lacanobia.oleracea* | Noctuidae | Lepidoptera | moth | 4% | 1% | 0% | 4% | 2% |
| *Orthosia.cerasi* | Noctuidae | Lepidoptera | moth | 4% | 1% | 3% | 0% | 2% |
| *Pardosa.amentata* | Lycosidae | Araneae | spider | 4% | 1% | 3% | 0% | 2% |
| *Pinus.sp.* | Pinaceae | Pinales | pine | 4% | 1% | 0% | 4% | 2% |
| *Quercus.robur* | Fagaceae | Fagales | English oak | 4% | 1% | 2% | 2% | 2% |
| *Spilosoma.lubricipedum* | Erebidae | Lepidoptera | moth | 4% | 1% | 0% | 4% | 2% |
| *Xestia.triangulum* | Noctuidae | Lepidoptera | moth | 4% | 1% | 2% | 2% | 2% |
| *Agrostis.sp.* | Poaceae | Poales | bentgrass | 0% | 1% | 2% | 0% | 1% |
| *Araneus.triguttatus* | Araneidae | Araneae | spider | 0% | 1% | 0% | 2% | 1% |
| *Avena.fatua* | Poaceae | Poales | wild oat | 0% | 1% | 2% | 0% | 1% |
| *Chalarus.fimbriatus* | Pipunculidae | Diptera | fly | 0% | 1% | 2% | 0% | 1% |
| *Crepidodera.fulvicornis* | Chrysomelidae | Coleoptera | beetle | 0% | 1% | 0% | 2% | 1% |
| *Dactylis.glomerata* | Poaceae | Poales | Cock’s-foot / cat grass | 0% | 1% | 0% | 2% | 1% |
| *Ditula.angustiorana* | Tortricidae | Lepidoptera | moth | 0% | 1% | 2% | 0% | 1% |
| *Drapetisca.socialis* | Linyphiidae | Araneae | spider | 0% | 1% | 0% | 2% | 1% |
| *Elachiptera.decipiens* | Chloropidae | Diptera | fly | 0% | 1% | 2% | 0% | 1% |
| *Embothrium.coccineum* | Proteaceae | Proteales | Chilean flame tree | 0% | 1% | 0% | 2% | 1% |
| *Epiphyas.postvittana* | Tortricidae | Lepidoptera | moth | 0% | 1% | 0% | 2% | 1% |
| *Forficula.sp.* | Forficulidae | Dermaptera | earwig | 0% | 1% | 2% | 0% | 1% |
| *Helosciadium.sp.* | Apiaceae | Apiales | water cress | 0% | 1% | 0% | 2% | 1% |
| *Hemerobius.micans* | Hemerobiidae | Neuroptera | lacewing | 0% | 1% | 2% | 0% | 1% |
| *Hepialus.fusconebulosus* | Hepialidae | Lepidoptera | moth | 0% | 1% | 2% | 0% | 1% |
| *Hordeum.vulgare* | Poaceae | Poales | barley | 0% | 1% | 0% | 2% | 1% |
| *Ligustrum.vulgare* | Oleaceae | Lamiales | privet | 0% | 1% | 2% | 0% | 1% |
| *Linum.usitatissimum* | Linaceae | Malpighiales | Linseed / flax | 0% | 1% | 0% | 2% | 1% |
| *Macrosiphum.funestum* | Aphididae | Hemiptera | aphid | 0% | 1% | 2% | 0% | 1% |
| *Melangyna.lasiophthalma* | Syrphidae | Diptera | hoverfly | 0% | 1% | 0% | 2% | 1% |
| *Meteorus.consimilis* | Braconidae | Hymenoptera | parasitoid wasp | 0% | 1% | 0% | 2% | 1% |
| *Neon.reticulatus* | Salticidae | Araneae | spider | 0% | 1% | 2% | 0% | 1% |
| *Neriene.peltata* | Linyphiidae | Araneae | spider | 0% | 1% | 0% | 2% | 1% |
| *Orthosia.gothica* | Noctuidae | Lepidoptera | moth | 0% | 1% | 0% | 2% | 1% |
| *Rhododendron.caucasicum* | Ericaceae | Ericales | rhododendron | 0% | 1% | 0% | 2% | 1% |
| *Rhopalosiphum.padi* | Aphididae | Hemiptera | aphid | 0% | 1% | 0% | 2% | 1% |
| *Scrophularia.balbisii* | Scrophulariaceae | Lamiales | figwort | 0% | 1% | 2% | 0% | 1% |
| *Spilarctia.luteum* | Erebidae | Lepidoptera | moth | 0% | 1% | 2% | 0% | 1% |
| *Syrphus.opinator* | Syrphidae | Diptera | hoverfly | 0% | 1% | 0% | 2% | 1% |
| *Syrrhizus.sp.* | Braconidae | Hymenoptera | parasitoid wasp | 0% | 1% | 2% | 0% | 1% |
| *Triticum.sp.* | Poaceae | Poales | wheat | 0% | 1% | 0% | 2% | 1% |
| *Umbilicus.horizontalis* | Crassulaceae | Saxifragales | Horizontal navelwort | 0% | 1% | 2% | 0% | 1% |
| *Veronica.persica* | Plantaginaceae | Lamiales | Birdeye speedwell | 0% | 1% | 0% | 2% | 1% |
| *Xestia.xanthographa* | Noctuidae | Lepidoptera | moth | 0% | 1% | 2% | 0% | 1% |
| *Calluna.vulgaris* | Ericaceae | Ericales | ling heather | 4% | 0% | 2% | 0% | 1% |
| *Coelichneumon.deliratorius* | Ichneumonidae | Hymenoptera | parasitoid wasp | 4% | 0% | 0% | 2% | 1% |
| *Elatobium.abietinum* | Aphididae | Hemiptera | aphid | 4% | 0% | 0% | 2% | 1% |
| *Galium.aparine* | Rubiaceae | Gentianales | stickyweed | 4% | 0% | 2% | 0% | 1% |
| *Hydriomena.furcata* | Geometridae | Lepidoptera | moth | 4% | 0% | 0% | 2% | 1% |
| *Orthosia.incerta* | Noctuidae | Lepidoptera | moth | 4% | 0% | 0% | 2% | 1% |
| *Phragmatobia.fuliginosa* | Erebidae | Lepidoptera | moth | 4% | 0% | 2% | 0% | 1% |
| *Quercus.macranthera* | Fagaceae | Fagales | Persian oak | 4% | 0% | 0% | 2% | 1% |
| *Rosa.sp.* | Rosaceae | Rosales | rose | 4% | 0% | 0% | 2% | 1% |
| *Sambucus.sp.* | Adoxaceae | Dipsacales | elderberry | 4% | 0% | 2% | 0% | 1% |
| *Syrphus.ribesii* | Syrphidae | Diptera | hoverfly | 4% | 0% | 2% | 0% | 1% |

Table S5. Univariate results from the *anova* test in the manyglm model. Results are taken from the full summary output. Data is from the full dataset but without any duplicate samples, or from the reduced dataset (only including species present in more than 10% of total samples). The only interaction that had any significant univariate effects was for age × sex in the full plant dataset, see model d. Species are highlighted according to higher prevalence with green for spring, 2017, deciduous, adult and female, and blue for winter, 2018, conifer, first year and male. Note that *Ochropleura implecta* is a North American species and is most likely to be the European *Ochropleura plecta* (flame shoulder moth) instead, though we do not know for certain.

| **Data set** | **Predictor** | **Prey Order** | **Prey Family** | **Prey Species** | **Group** | **Common name** | **LRT** | **P** |
| --- | --- | --- | --- | --- | --- | --- | --- | --- |
| a) Invertebrates year round; full | Season  (Spring is green, winter is blue) | Lepidoptera  Lepidoptera  Araneae  Lepidoptera  Lepidoptera  Lepidoptera  Diptera  Araneae  Araneae  Araneae  Diptera  Lepidoptera  Lepidoptera  Hemiptera  Hymenoptera  Lepidoptera  Hymenoptera  Lepidoptera  Diptera  Diptera  Diptera  Lepidoptera  Hymenoptera  Lepidoptera  Lepidoptera  Lepidoptera  Lepidoptera  Lepidoptera  Lepidoptera  Diptera  Hymenoptera  Hymenoptera  Lepidoptera  Lepidoptera  Diptera  Diptera  Lepidoptera | Geometridae  Noctuidae  Amaurobiidae  Noctuidae  Noctuidae  Tortricidae  Syrphidae  Clubionidae  Clubionidae  Clubionidae  Ceratopogonidae  Noctuidae  Erebidae  Aphididae  Ichneumonidae  Tortricidae  Braconidae  Geometridae  Lonchopteridae  Syrphidae  Syrphidae  Noctuidae  Cynipidae  Tortricidae  Noctuidae  Geometridae  Noctuidae  Noctuidae  Tortricidae  Muscidae  Ichneumonidae  Ichneumonidae  Noctuidae  Tortricidae  Scathophagidae  Syrphidae  Geometridae | *Agriopis marginaria*  *Agrochola lota*  *Amaurobius fenestralis*  *Amphipyra pyramidea*  *Anorthoa munda*  *Archips podana*  *Cheilosia semifasciata*  *Clubiona comta*  *Clubiona reclusa*  *Clubiona terrestris*  *Culicoides impunctatus*  *Dryobotodes eremita*  *Eilema depressum*  *Elatobium abietinum*  *Enytus montanus*  *Epinotia nisella*  *Glyptapanteles porthetriae*  *Hydriomena furcata*  *Lonchoptera lutea*  *Melangyna lasiophthalma*  *Meliscaeva auricollis*  *Mesapamea secalella*  *Neuroterus quercusbaccarum*  *Notocelia uddmanniana*  *Ochropleura implecta*  *Operophtera brumata*  *Orthosia cerasi*  *Orthosia cruda*  *Pandemis cerasana*  *Phaonia tuguriorum*  *Phobocampe bicingulata*  *Plectiscus impurator*  *Polia nebulosa*  *Ptycholoma lecheana*  *Scathophaga stercoraria*  *Syrphus ribesii*  *Thera britannica* | Moth  Moth  Spider  Moth  Moth  Moth  Hoverfly  Sac spider  Sac spider  Sac spider  Midge  Moth  Moth  Aphid  Parasitoid wasp  Moth  Parasitoid wasp  Moth  Fly  Hoverfly  Hoverfly  Moth  Gall wasp  Moth  Moth  Moth  Moth  Moth  Moth  Fly  Parasitoid wasp  Parasitoid wasp  Moth  Moth  Fly  Hoverfly  Moth | Dotted border  Red-line quaker  Lace-web spider  Copper underwing  Twin-spotted quaker  Large fruit-tree tortrix  -  -  -  -  Highland midge  Brindled green  Buff footman  Green spruce aphid  -  Grey poplar bell  -  July Highflyer  Yellow spear-winged fly  -  -  Less common rustic  Common spangle gall wasp  Bramble shoot  Flame shoulder (dart)  Winter moth  Common quaker  Small quaker  Barred fruit-tree tortrix  -  -  -  Grey arches  Leche's twist moth  Yellow dung fly  -  Spruce carpet | 20.7  18.3  18.7  28.3  28.5  11.1  11.1  18.7  23.3  16.7  12.3  14.6  23.3  21.5  12.3  19.5  12.3  27.9  14.8  16.7  17.0  11.1  33.0  15.8  14.8  112.5  55.7  17.0  35.3  12.9  12.3  11.0  12.9  14.6  15.8  19.1  17.0 | 0.001  0.001  0.001  0.001  0.001  0.04  0.04  0.001  0.001  0.002  0.03  0.005  0.001  0.001  0.03  0.001  0.03  0.001  0.003  0.001  0.001  0.04  0.001  0.002  0.002  0.001  0.001  0.001  0.001  0.03  0.03  0.06  0.02  0.005  0.002  0.001  0.001 |
| b) Invertebrates in spring; full | Habitat  (Deciduous is green, conifer is blue) | Lepidoptera  Hemiptera  Lepidoptera  Diptera  Lepidoptera | Noctuidae  Aphididae  Geometridae  Syrphidae  Noctuidae | *Agrochola lota*  *Elatobium abietinum*  *Hydriomena furcata*  *Meliscaeva auricollis*  *Orthosia cerasi* | Moth  Aphid  Moth  Hoverfly  Moth | Red-line quaker  Green spruce aphid  July highflyer  -  Common quaker | 20.7  13.4  12.4  13.4  12.4 | 0.001  0.02  0.02  0.02  0.02 |
|  | Year  (2017 is green, 2018 is blue) | Araneae  Hymenoptera  Hymenoptera  Diptera  Diptera  Diptera | Clubionidae  Ichneumonidae  Braconidae  Syrphidae  Syrphidae  Syrphidae | *Clubiona reclusa*  *Enytus montanus*  *Glyptapanteles porthetriae*  *Meliscaeva auricollis*  *Syrphus ribesii*  *Syrphus torvus* | Sac spider  Parasitoid wasp  Parasitoid wasp  Hoverfly  Hoverfly  Hoverfly | -  -  -  -  -  Hairy-eyed flower fly | 15.6  12.3  12.3  23.7  14.4  13.4 | 0.004  0.05  0.05  0.001  0.01  0.02 |
|  | Sex  (Female is green, male is blue) | Hymenoptera  Hymenoptera  Diptera | Ichneumonidae  Braconidae  Psychodidae | *Enytus montanus*  *Glyptapanteles porthetriae*  *Psychoda trinodulosa* | Parasitoid wasp  Parasitoid wasp  Moth fly | -  -  - | 13.1  13.1  12.6 | 0.04  0.04  0.05 |
|  | Age | None | None | None | None | - | - | - |
| c) Invertebrates in spring; excluding species in <10% of samples | Habitat  (Deciduous is green, conifer is blue) | Lepidoptera  Hemiptera  Lepidoptera  Diptera  Diptera  Lepidoptera  Diptera | Noctuidae  Aphididae  Geometridae  Syrphidae  Syrphidae  Noctuidae  Syrphidae | *Agrochola.lota*  *Elatobium.abietinum*  *Hydriomena.furcata*  *Melangyna lasiophthalma*  *Meliscaeva.auricollis*  *Orthosia.cerasi*  *Syrphus.ribesii* | Moth  Aphid  Moth  Hoverfly  Hoverfly  Moth  Hoverfly | Red-line quaker  Green spruce aphid  July highflyer  -  -  Common quaker  - | 21.4  16.3  13.6  10.9  20.9  12.3  13.0 | 0.001  0.004  0.01  0.04  0.002  0.02  0.02 |
|  | Year | Lepidoptera  Araneae  Hymenoptera  Hymenoptera  Diptera  Diptera  Lepidoptera | Geometridae  Clubionidae  Ichneumonidae  Braconidae  Syrphidae  Syrphidae  Geometridae | *Agriopis marginaria*  *Clubiona.reclusa*  *Enytus montanus*  *Glyptapanteles porthetriae*  *Meliscaeva.auricollis*  *Syrphus.ribesii*  *Thera britannica* | Moth  Sac spider  Parasitoid wasp  Parasitoid wasp  Hoverfly  Hoverfly  Moth | Dotted border  -  -  -  -  -  Spruce carpet | 11.0  17.3  13.0  13.0  14.1  10.8  11.0 | 0.04  0.003  0.02  0.02  0.01  0.05  0.04 |
|  | Sex  (Female is green, male is blue) | Hymenoptera  Hymenoptera  Lepidoptera | Ichneumonidae  Braconidae  Geometridae | *Enytus.montanus*  *Glyptapanteles.porthetriae*  *Thera.britannica* | Parasitoid wasp  Parasitoid wasp  Moth | -  -  Spruce carpet | 13.4  13.4  10.9 | 0.02  0.02  0.05 |
|  | Age | None | None | None | None | - | - | - |
| d) Plants and invertebrates in winter; full | Habitat | Fagales | Fagaceae | *Fagus sp.* | Beech | Beech | 15.9 | 0.002 |
|  | Age  (Adult is green, first year is blue) | Fagales | Fagaceae | *Fagus sp.* | Beech | Beech | 13.3 | 0.009 |
|  | Sex | None | None | None | None | - | - | - |
|  | Age x Sex  (Adult female is green) | Diptera | Muscidae | *Phaonia tuguriorum* | Fly | - | 16.7 | 0.01 |
| e) Plants and invertebrates in winter; excluding species in <10% of samples | Habitat | Fabales  Fagales  Hymenoptera | Fabaceae  Fagaceae  Cynipidae | *Arachis.sp.*  *Fagus.sp.*  *Neuroterus quercusbaccarum* | Peanut  Beech  Gall wasp | Peanut  Beech  Common spangle gall wasp | 11.8  10.0  6.61 | 0.002  0.004  0.06 |
|  | Age  (Adult is green, first year is blue) | Fagales | Fagaceae | *Fagus.sp.* | Beech | Beech | 12.0 | 0.005 |
|  | Sex | None | None | None | None | - | - | - |
